# Describing the emotional exhaustion, depersonalization, and low personal accomplishment symptoms associated with Maslach Burnout Inventory subscale scores in US physicians: an item response theory analysis

**DOI:** 10.1186/s41687-020-00204-x

**Published:** 2020-06-01

**Authors:** Keri J. S. Brady, Pengsheng Ni, R. Christopher Sheldrick, Mickey T. Trockel, Tait D. Shanafelt, Susannah G. Rowe, Jeffrey I. Schneider, Lewis E. Kazis

**Affiliations:** 1 Health Law, Policy & Management Department, Boston University School of Public Health, 715 Albany Street, Boston, MA USA; 2 Biostatistics & Epidemiology Data Analytic Center, Boston University School of Public Health, 85 East Newton Street, Boston, MA USA; 3 Department of Psychiatry and Behavioral Sciences, Stanford University, 401 Quarry Road, Stanford, CA USA; 4 Stanford Medicine WellMD Center, Stanford University, 300 Pasteur Drive, Suite H3215, Stanford, CA USA; 5 Boston Medical Center, 1 Boston Medical Center Place, Boston, MA USA; 6 Department of Ophthalmology, Boston University School of Medicine, 85 East Concord Street, 8th Floor, Boston, MA USA; 7 Department of Emergency Medicine, Boston University School of Medicine, 72 East Concord Street, Boston, MA USA

**Keywords:** Physician burnout, Physician well-being, Burnout measurement, Health outcome measurement, Person-centered outcome measurement

## Abstract

**Purpose:**

Current US health policy discussions regarding physician burnout have largely been informed by studies employing the Maslach Burnout Inventory (MBI); yet, there is little in the literature focused on interpreting MBI scores. We described the burnout symptoms and precision associated with MBI scores in US physicians.

**Methods:**

Using item response theory (IRT) analyses of secondary, cross-sectional survey data, we created response profiles describing the probability of burnout symptoms associated with US physicians’ MBI emotional exhaustion (EE), depersonalization (DP), and personal accomplishment (PA) subscale scores. Response profiles were mapped to raw subscale scores and used to predict symptom endorsements at mean scores and commonly used cut-points.

**Results:**

The average US physician was likely to endorse feeling he/she is emotionally drained, used up, frustrated, and working too hard and all PA indicators once weekly or more but was unlikely to endorse feeling any DP symptoms once weekly or more. At the commonly used EE and DP cut-points of 27 and 10, respectively, a physician was unlikely to endorse feeling burned out or any DP symptoms once weekly or more. Each subscale assessed the majority of sample score ranges with ≥ 0.70 reliability.

**Conclusions:**

We produced a crosswalk mapping raw MBI subscale scores to scaled scores and response profiles calibrated in a US physician sample. Our results can be used to better understand the meaning and precision of MBI scores in US physicians; compare individual/group MBI scores against a reference population of US physicians; and inform the selection of subscale cut-points for defining categorical physician burnout outcomes.

## Introduction

Current US health policy discussions surrounding the physician burnout crisis have largely been informed by prevalence studies employing the Maslach Burnout Inventory-Human Services Survey for Medical Personnel (MBI) [1–9]. While the MBI is the most widely used physician burnout outcome assessment, a recent systematic review found a lack of consistency in cut-points used to define dichotomous burnout outcomes on each continuous MBI subscale [8], contributing to a marked heterogeneity in reported burnout prevalences across studies.

One contributor to the observed inconsistencies in defining dichotomous burnout outcomes on the MBI may be the lack of clarity regarding the meaning of subscale scores. Traditional measurement methods do not permit users to directly compare subscale scores with the content of items to interpret their meaning. The use of item response theory (IRT) measurement methods can facilitate an enhanced understanding of subscale scores over traditional methods [10, 11]. Using IRT to estimate physicians’ probability of endorsing MBI subscale items across different burnout symptom severity levels, scores can be interpreted based on how likely a physician is to endorse a particular item (e.g., “I feel burned out from my work”) at a particular frequency (e.g., “once a week” or more) and relative to the mean score of the sample (i.e., content-referenced and norm-referenced scoring, respectively). IRT analyses are routinely used in health outcome measurement and are part of the NIH Patient Reported Outcome Measurement Information System (PROMIS) scientific standards for health outcome measurement development and validation [12]. However, no studies have applied IRT methods to evaluate the MBI in a national sample of US physicians.

In this study, we leveraged the content-referenced and norm-referenced score interpretation of IRT-calibrated (estimated) models to better understand the meaning of MBI subscale scores in a national US physician sample. Our primary aim was to create response profiles describing the probability of burnout symptoms across standardized MBI subscale scores in US physicians. We produced a crosswalk mapping raw (total) MBI subscale scores to scaled (IRT-based) scores and associated response profiles. As a secondary aim, we evaluated the precision bandwidth of each MBI subscale relative to where US physicians’ scores are distributed on each metric.

## Methods

### Design and sample

This study used secondary survey data on the 22-item MBI from the 2014 wave of the anonymous [1, 2], cross-sectional study conducted by Shanafelt et al. (2015) to monitor the national prevalence of physician burnout [4]. Participants were sampled via email from the American Medical Association Physician Master File. Further sampling design details are published in Shanafelt et al. (2015) [4]. From this dataset, we excluded physicians who were not in practice in the US or retired at the time of the survey.

### Measures

The MBI is a measure of job burnout defined by three subscales: emotional exhaustion (EE) (9 items), depersonalization (DP) (5 items), and professional accomplishment (PA) (8 items), each with 7-point Likert-type, frequency response scale (0 = never, 1 = a few times a year or less, 2 = once a month or less, 3 = a few times a month, 4 = once a week, 5 = a few times a week, 6 = every day) [1, 2]. Scales are scored such that higher scores indicate more of each construct. Higher scores on the EE and DP subscales indicate a higher burnout symptom burden; lower scores on the PA subscale indicate a higher burnout symptom burden.

### Statistical analyses

Our analytic approach was informed by the PROMIS scientific standards for instrument development and validation [12].

#### IRT model calibration

We calibrated IRT models for each MBI subscale using unidimensional, graded response models (GRM) [13]. For each MBI subscale item, the GRM predicted the cumulative probability of responding in a particular item response category or higher (e.g., “once a week” to “every day”) as a function of physicians’ underlying (latent) burnout symptom levels (i.e., an IRT score (*θ*)), item threshold parameters (${b}_{x_j}$), and an item discrimination parameter (*a*_*j*_). Item threshold parameters represent the IRT score at which a randomly selected physician among those with that score would have a cumulative probability of endorsing a particular response category or higher of 0.50. The mean of item threshold estimates from each calibrated IRT model describe the burnout symptom severity (item difficulty) represented by each item. IRT scores, item threshold parameters, and item symptom severity values are on z-score metric (0 = mean, SD = 1). Item discrimination parameters indicate the degree to which an item differentiates between physicians who have high versus low burnout symptom levels (with higher values yielding more scale precision). The GRM model assumes that physicians’ item responses are a function of one primary, continuous underlying construct (unidimensionality); item responses are independent after controlling for the underlying burnout construct (local independence); and the probability of endorsing successively higher item response categories increases as physicians’ underlying burnout symptom levels increase (monotonicity) [14]. Prior to calibrating each IRT model, we evaluated traditional item- and scale-level descriptive statistics; IRT model assumptions; and model-, item-, and person-level fit (Supplemental Appendix 1) [12].

#### Response profiles

To describe the severity of burnout symptoms associated with MBI subscale scores, we created response profiles from each calibrated IRT model that predict the cumulative probability that a randomly selected physician endorses each item (i.e., symptom) at a frequency of *“once a week” or more* (i.e., “a few times a week” or “every day”) across IRT-based subscale scores. We selected a frequency of *“once a week” or more* for each response profile as this is commonly used as the frequency for defining burnout in national prevalence studies [5, 15, 16]. To enable instrument users to interpret individuals’/groups’ MBI subscale scores in relation to response profiles, we created a crosswalk mapping raw (total) subscale scores to IRT-based z-scores and associated standard errors (SEs) using expected a posteriori (EAP) sum scoring [17]. The crosswalks and associated response profiles allow instrument users to interpret individuals’/groups’ scores relative to how likely a randomly selected physician among those with the particular score is likely to endorse each item at a frequency of “once a week” or more. We also present IRT-based t-scores (mean = 50, SD = 10) in each crosswalk. To illustrate how each response profile can be used, we interpreted the response profiles for z-scores at or nearest to the mean and at commonly used cut-points for defining dichotomous burnout outcomes on each subscale (≥ 27, ≥ 10, and ≤ 33 on the EE, DP, and PA subscales, respectively) [8]. In our interpretation of the burnout symptom severity associated with mean subscale scores and commonly used subscale cut-points, we defined an item as *likely to be endorsed* or *not likely to be endorsed* if it had a respective > 0.50 or < 0.50 cumulative probability of endorsement (response probability criterion) at a particular z-score.

#### Precision bandwidth

We used test information functions (TIFs) to evaluate each subscale’s precision bandwidth by assessing whether each metric demonstrated adequate reliability for group- and individual-level measurement where sample scores (computed using EAP scoring) are distributed. A TIF describes the precision of a scale across z-scores and is inversely related to a scale’s standard error (SE) [14]. Higher information equates to more reliability and lower SE associated with an individual’s/group’s subscale score. Adequate reliability for group- and individual-level measurement was defined as 0.70 and 0.90, respectively [12].

All statistical analyses were conducted in R (v3.5.1) using the *psych* (v1.8.12) [18, 19], *lavaan* (v0.6–3) [20], and *mirt* packages (v1.30.6) [21]. This study was approved by the Boston University Medical Campus Institutional Review Board (approval # H-37414).

## Results

The overall sample included 6682 multi-specialty US physicians (Table 1). The majority of the sample was male and a non-primary care physician.

**Table 1:** MBI Overall Sample Characteristics (*n* = 6682)

Characteristic	n (%) ^a^
Sex	
Male	4346 (65.0)
Female	2123 (32.8)
Missing	213 (3.2)
Primary Care	
Primary care	1559 (23.3)
Non-primary care	5089 (76.2)
Missing	34 (0.5)
Specialty	
Anesthesiology	231 (3.5)
Dermatology	163 (2.4)
Emergency Medicine	348 (5.2)
Family Medicine	520 (7.8)
General Surgery	246 (3.7)
General surgery subspecialty	370 (5.5)
General Internal Medicine	447 (6.7)
General Pediatrics	356 (5.3)
Internal Medicine-subspecialty	759 (11.4)
Neurology	239 (3.6)
Neurosurgery	56 (0.8)
Obstetrics and gynecology	284 (4.3)
Ophthalmology	232 (3.5)
Orthopedic surgery	234 (3.5)
Otolaryngology	161 (2.4)
Other	231 (3.5)
Pathology	168 (2.5)
Pediatric subspecialty	311 (4.7)
Physical medicine and rehabilitation	170 (2.5)
Preventive medicine, occupational medicine, or environmental medicine	106 (1.6)
Psychiatry	551 (8.2)
Radiation oncology	64 (1.0)
Radiology	255 (3.8)
Urology	116 (1.7)
Missing	64 (1.0)

^a^*Percentages do not add to 100 due to rounding*

### IRT calibration

The final calibrated EE, DP, and PA IRT models (Table 2) achieved adequate model-data fit and met all model assumptions (Supplemental Appendix 2) [22]. However, items DP4, PA2, and PA5 showed a lack of monotonicity across one or more adjacent response category pairs and items EE4 (working with people all day is a real strain) and EE8 (working with people directly puts too much stress on me) showed local dependence. While the former violation can be resolved by collapsing adjacent, non-monotonic item response categories [12], we chose to maintain the original scoring of the subscales and the ability to interpret published subscale scores relative to response profiles. Sensitivity analyses of DP and PA calibrations with and without collapsed item response categories showed minimal differences in item parameter estimates. The latter violation was remedied by summing the EE4 and EE8 items to form one scale (coded 0 to 12). The combined EE4EE8 item was used in the final calibrated EE IRT model in place of the individual items.

**Table 2:** Item Parameter Estimates and Standard Errors (SE) for Calibrated Emotional Exhaustion (EE), Depersonalization (DP), and Personal Accomplishment (PA) IRT Models ^a^

		Item Discrimination Estimates	Item Threshold Estimates and SEs						
IRT Model (n)	Item	*a*_*j*_ (se)	${b}_{1_j}$ (se)	${b}_{2_j}$ (se)	${b}_{3_j}$ (se)	${b}_{4_j}$ (se)	${b}_{5_j}$ (se)	${b}_{6_j}$ (se)	Item Symptom Severity ^b^
**EE**^c^ (*n* = 6264)	EE1: emotionally drained	4.32 (.09)	−1.84 (0.03)	− 1.02 (0.02)	−0.60 (0.02)	− 0.07 (0.02)	0.29 (0.02)	1.19 (0.02)	−0.34
	EE2: used up	3.92 (.08)	−1.83 (0.03)	−1.17 (0.02)	−0.75 (0.02)	− 0.26 (0.02)	0.09 (0.02)	0.98 (0.02)	−0.49
	EE3: fatigued	3.14 (.06)	−1.33 (0.03)	−0.70 (0.02)	−0.27 (0.02)	0.20 (0.02)	0.57 (0.02)	1.39 (0.03)	−0.02
	EE5: burned out	4.36 (.09)	−1.18 (0.02)	−0.44 (0.02)	−0.10 (0.02)	0.27 (0.02)	0.60 (0.02)	1.23 (0.02)	0.07
	EE6: frustrated	2.73 (.05)	−1.98 (0.04)	−1.09 (0.02)	−0.67 (0.02)	−0.18 (0.02)	0.20 (0.02)	1.02 (0.02)	−0.45
	EE7: working too hard	2.44 (.05)	−1.71 (0.03)	−1.08 (0.02)	−0.66 (0.02)	−0.18 (0.02)	0.18 (0.02)	0.97 (0.02)	−0.41
	EE9: end of rope	2.43 (.05)	−0.41 (0.02)	0.31 (0.02)	0.65 (0.02)	1.00 (0.02)	1.34 (0.03)	2.06 (0.04)	0.83
**DP** (*n* = 6403)	DP1: treat patients as objects	1.76 (.05)	−0.02 (0.02)	0.73 (0.02)	1.18 (0.03)	1.64 (0.04)	2.08 (0.05)	3.31 (0.09)	1.49
	DP2: more callous	3.98 (.13)	−0.52 (0.02)	0.19 (0.02)	0.54 (0.02)	0.92 (0.02)	1.26 (0.02)	2.03 (0.04)	0.74
	DP3: job hardening me	2.67 (.06)	−0.55 (0.02)	0.12 (0.02)	0.46 (0.02)	0.78 (0.02)	1.11 (0.02)	1.64 (0.03)	0.59
	DP4: don’t care	1.56 (.05)	0.49 (0.02)	1.32 (0.04)	1.83 (0.05)	2.27 (0.06)	2.71 (0.07)	3.62 (0.11)	2.04
	DP5: patients blame me	1.07 (.03)	−1.70 (0.05)	−0.21 (0.03)	0.44 (0.03)	1.07 (0.04)	1.69 (0.05)	2.98 (0.09)	0.71
**PA** (*n* = 6201)	PA1: easily understand patients	1.09 (.04)	−4.95 (0.20)	−4.22 (0.15)	−3.66 (0.13)	−2.73 (0.09)	−2.15 (0.07)	−0.80 (0.04)	−3.08
	PA2: deal effectively with patient problems	1.63 (.05)	−3.49 (0.11)	−3.16 (0.09)	− 2.91 (0.08)	− 2.44 (0.06)	−1.98 (0.05)	−0.79 (0.03)	− 2.46
	PA3: positively influencing others	2.55 (.07)	−2.97 (0.08)	−2.46 (0.05)	− 2.08 (0.04)	−1.54 (0.03)	−1.14 (0.03)	−0.24 (0.02)	− 1.74
	PA4: feel energetic	1.45 (.04)	−3.09 (0.08)	−2.49 (0.06)	−1.80 (0.04)	−0.94 (0.03)	−0.38 (0.02)	1.25 (0.04)	−1.24
	PA5: can create relaxed atmosphere	1.88 (.05)	−2.91 (0.08)	−2.54 (0.06)	− 2.26 (0.05)	−1.79 (0.04)	−1.43 (0.03)	−0.40 (0.02)	− 1.89
	PA6: exhilarated	1.95 (.05)	−2.54 (0.06)	−1.89 (0.04)	−1.40 (0.03)	−0.76 (0.02)	−0.29 (0.02)	0.93 (0.03)	−0.99
	PA7: accomplished many things	2.53 (.07)	−3.13 (0.09)	−2.28 (0.05)	−1.77 (0.04)	−1.22 (0.03)	−0.79 (0.02)	0.19 (0.02)	−1.50
	PA8: deal with problems calmly	1.21 (.04)	−4.88 (0.19)	−3.83 (0.13)	−3.05 (0.09)	−2.19 (0.06)	−1.54 (0.05)	−0.10 (0.03)	−2.60

^a^Higher scores on each scale indicate more of each construct; higher scores on the EE and DP scales indicate more burnout symptoms; lower scores on the PA scale indicate more burnout symptoms. “ a_j_ ” parameter for the EE, DP, and PA IRT models = item discrimination parameter estimate, which indicates the degree to which an item discriminates between physicians with high versus low underlying EE, DP, or PA levels. Higher discrimination estimates indicate that the item is more discriminating compared to items with lower discrimination estimates. Item threshold estimates (${\mathrm{b}}_{1_{\mathrm{j}}}$ to ${\mathrm{b}}_{6_{\mathrm{j}}}$) indicate the IRT score at which a randomly selected physician among those with that score would have a 50% chance of endorsing the particular response category or a higher response category. For items in each model: “ ${\mathrm{b}}_{1_{\mathrm{j}}}$ ” = threshold parameter for endorsing “few times a year or less” or more; “ ${\mathrm{b}}_{2_{\mathrm{j}}}$ ” = threshold parameter estimate for endorsing “once a month or less” or more; “ ${\mathrm{b}}_{3_{\mathrm{j}}}$ ” = threshold parameter estimate for endorsing “a few times a month” or more; “ ${\mathrm{b}}_{4_{\mathrm{j}}}$ ” = threshold parameter estimate for endorsing “once a week” or more; “ ${\mathrm{b}}_{5_{\mathrm{j}}}$ ” = threshold parameter estimate for endorsing “a few times a week” or more; “ ${\mathrm{b}}_{6_{\mathrm{j}}}$ ” = threshold parameter estimate for endorsing “every day”. ^b^ Item symptom severity is the mean of item threshold parameter estimates (i.e., item difficulty). On the EE and DP subscales, items with lower item symptom severity values indicate that an item is easier to endorse and represents less severe burnout symptoms; higher item symptom severity values indicate that the item is harder to endorse and represents more severe burnout symptoms. On the PA subscale, items with lower symptom severity values indicate an item is harder to endorse and represents more severe burnout symptoms; items with higher symptom severity values indicate the item is easier to endorse and represents less severe burnout symptoms. ^c^ Item parameter estimates and associated SEs for the combined EE4EE8 item included in the EE IRT model are: a = 1.52 (.03); ${\mathrm{b}}_{1_{\mathrm{j}}}$ = − 1.23 (.03), ${\mathrm{b}}_{2_{\mathrm{j}}}$ = − 0.68 (.03), ${\mathrm{b}}_{3_{\mathrm{j}}}$ = − 0.13 (.02), ${\mathrm{b}}_{4_{\mathrm{j}}}$ = 0.28 (.02), ${\mathrm{b}}_{5_{\mathrm{j}}}$ = 0.63 (.03), ${\mathrm{b}}_{6_{\mathrm{j}}}$ = 0.96 (.03), ${\mathrm{b}}_{7_{\mathrm{j}}}$ = 1.31 (.03), ${\mathrm{b}}_{8_{\mathrm{j}}}$ = 1.57 (.04), ${\mathrm{b}}_{9_{\mathrm{j}}}$ = 1.90 (.05), ${\mathrm{b}}_{10_{\mathrm{j}}}$ = 2.23 (.05), ${\mathrm{b}}_{11_{\mathrm{j}}}$ = 2.76 (.07), ${\mathrm{b}}_{12_{\mathrm{j}}}$ = 3.35 (.09). Item symptom severity for EE4EE8 = 1.08

### Item symptom severity

The least severe burnout symptoms (Table 2) include: feeling used up (EE2), feeling emotionally hardened (DP3), and lacking feelings of exhilaration after working closely with patients (PA6). Whereas, the most severe burnout symptoms include: feeling that working with people is a real strain/too much stress (EE4EE8), not really caring what happens to some patients (DP4), and not easily understanding how patients feel (PA1).

### Response profiles

#### Emotional exhaustion subscale

A physician scoring approximately at the mean (raw score of 26) on the EE subscale is likely to endorse feeling emotionally drained from work (EE1), used up at the end of the workday (EE2), frustrated from his/her job (EE6), and that he/she is working too hard on his/her job (EE7) at a frequency of once weekly or more (Table 3; see Supplemental Appendix 3 for plotted cumulative probability curves and option response functions). A physician from this latent EE level would, however, be unlikely to report feeling: fatigued when getting up and having to face another day on the job (EE3), burned out from work (EE5), that working with people is stressful/straining (EE4EE8), or at the end of his/her rope (EE9) once weekly or more. The commonly used raw score cut-point of 27 on the EE subscale corresponds to a z-score that is 0.07 SDs above the mean EE level of US physicians. At this score, a randomly selected physician would be likely to report feeling the same EE symptoms as a physician scoring at the mean. Endorsing feeling fatigued (EE3), burned out (EE5), at the end of your rope (EE9), and working with people is too stressful/straining (EE4EE8) once weekly or more is likely among physicians with z-scores > 0.20, > 0.27, > 1.00, > 1.57 SDs above the mean, respectively.

**Table 3:** Crosswalks mapping raw (total) Emotional Exhaustion (EE), Depersonalization (DP), and Personal Accomplishment (PA) subscale scores to IRT-based scores and response profiles

**Raw (total) scores and corresponding IRT-scores**^*a*^			**Probability of a US physician endorsing each item at a frequency of “once a week” or more**							
*EE raw score*	*EE IRT* *z-score (θ) (se)*	*EE IRT t-score (se)*	***EE items***^***b***^							
			*EE1: emotionally drained from work* ${P}_{4_{EE1}}$*(θ)*	*EE2: used up at end of workday* ${P}_{4_{EE2}}$*(θ)*	*EE3: fatigued when get up* ${P}_{4_{EE3}}$*(θ)*	*EE5: burned out from work* ${P}_{4_{EE5}}$*(θ)*	*EE6: frustrated by job* ${P}_{4_{EE6}}$*(θ)*	*EE7: working too hard on job* ${P}_{4_{EE7}}$*(θ)*	*EE4EE8: working with people is a real strain and/or puts too much stress on me* ${P}_{8_{EE4 EE8}}$*(θ)*	*EE9: at the end of my rope* ${P}_{4_{EE9}}$*(θ)*
0	−2.51 (0.43)	24.92 (4.26)	0.000	0.000	0.000	0.000	0.002	0.003	0.002	0.000
1	−2.13 (0.32)	28.72 (3.21)	0.000	0.001	0.001	0.000	0.005	0.008	0.004	0.000
2	−1.91 (0.30)	30.92 (2.95)	0.000	0.002	0.001	0.000	0.009	0.014	0.005	0.001
3	−1.74 (0.28)	32.58 (2.82)	0.001	0.003	0.002	0.000	0.014	0.021	0.006	0.001
4	−1.60 (0.27)	33.96 (2.72)	0.001	0.005	0.003	0.000	0.020	0.030	0.008	0.002
5	−1.49 (0.27)	35.14 (2.67)	0.002	0.008	0.005	0.000	0.027	0.039	0.009	0.002
6	−1.38 (0.26)	36.20 (2.62)	0.003	0.012	0.007	0.001	0.036	0.050	0.011	0.003
7	−1.28 (0.26)	37.19 (2.58)	0.005	0.018	0.009	0.001	0.047	0.063	0.013	0.004
8	−1.19 (0.26)	38.10 (2.55)	0.008	0.026	0.012	0.002	0.059	0.077	0.015	0.005
9	−1.10 (0.25)	38.96 (2.53)	0.012	0.036	0.016	0.003	0.075	0.095	0.017	0.006
10	−1.02 (0.25)	39.78 (2.51)	0.016	0.049	0.021	0.004	0.091	0.113	0.019	0.007
11	−0.94 (0.25)	40.56 (2.49)	0.023	0.066	0.027	0.005	0.111	0.134	0.021	0.009
12	−0.87 (0.25)	41.30 (2.47)	0.031	0.084	0.033	0.007	0.131	0.155	0.024	0.010
13	−0.80 (0.25)	42.02 (2.45)	0.041	0.108	0.041	0.009	0.155	0.179	0.026	0.012
14	−0.73 (0.24)	42.71 (2.44)	0.055	0.138	0.051	0.012	0.181	0.205	0.029	0.015
15	−0.66 (0.24)	43.38 (2.43)	0.073	0.174	0.062	0.017	0.211	0.235	0.032	0.017
16	−0.60 (0.24)	44.04 (2.42)	0.121	0.259	0.091	0.029	0.277	0.296	0.035	0.024
17	−0.53 (0.24)	44.68 (2.41)	0.151	0.307	0.107	0.038	0.311	0.328	0.039	0.027
18	−0.47 (0.24)	45.30 (2.41)	0.187	0.359	0.127	0.048	0.347	0.361	0.043	0.031
19	−0.41 (0.24)	45.92 (2.40)	0.230	0.414	0.149	0.062	0.385	0.395	0.047	0.036
20	−0.35 (0.24)	46.53 (2.40)	0.279	0.472	0.175	0.079	0.424	0.431	0.051	0.042
21	−0.29 (0.24)	47.13 (2.40)	0.334	0.531	0.204	0.100	0.465	0.467	0.055	0.048
22	−0.23 (0.24)	47.73 (2.41)	0.344	0.541	0.209	0.104	0.471	0.473	0.060	0.049
23	−0.17 (0.24)	48.32 (2.41)	0.394	0.589	0.236	0.127	0.505	0.504	0.066	0.055
24	−0.11 (0.24)	48.91 (2.42)	0.457	0.644	0.271	0.158	0.546	0.540	0.072	0.063
25	−0.05 (0.24)	49.51 (2.44)	0.522	0.696	0.310	0.196	0.587	0.576	0.078	0.072
**26**	**0.01 (0.25)**	**50.10 (2.45)**	**0.565**	**0.728**	**0.338**	**0.225**	**0.613**	**0.600**	**0.082**	**0.079**
27	0.07 (0.25)	50.70 (2.48)	0.647	0.786	0.396	0.292	0.663	0.646	0.092	0.094
28	0.13 (0.25)	51.30 (2.50)	0.704	0.823	0.442	0.349	0.699	0.679	0.100	0.107
29	0.19 (0.25)	51.90 (2.53)	0.755	0.854	0.489	0.411	0.732	0.710	0.109	0.122
30	0.25 (0.26)	52.51 (2.57)	0.800	0.881	0.536	0.475	0.763	0.739	0.118	0.139
31	0.31 (0.26)	53.13 (2.61)	0.838	0.904	0.582	0.540	0.791	0.766	0.128	0.157
32	0.37 (0.26)	53.75 (2.65)	0.870	0.922	0.627	0.604	0.817	0.792	0.138	0.177
33	0.44 (0.27)	54.38 (2.69)	0.901	0.940	0.677	0.674	0.844	0.818	0.151	0.204
34	0.50 (0.27)	55.01 (2.74)	0.922	0.952	0.717	0.729	0.864	0.839	0.163	0.228
35	0.57 (0.28)	55.65 (2.80)	0.941	0.963	0.759	0.785	0.885	0.861	0.178	0.260
36	0.63 (0.29)	56.30 (2.85)	0.954	0.971	0.792	0.826	0.901	0.878	0.192	0.289
37	0.70 (0.29)	56.96 (2.92)	0.965	0.977	0.826	0.866	0.917	0.895	0.209	0.325
38	0.76 (0.30)	57.64 (2.99)	0.973	0.982	0.851	0.893	0.928	0.908	0.225	0.358
39	0.83 (0.31)	58.34 (3.07)	0.980	0.986	0.877	0.919	0.940	0.921	0.244	0.398
40	0.91 (0.32)	59.06 (3.17)	0.986	0.990	0.902	0.942	0.951	0.934	0.267	0.445
41	0.98 (0.33)	59.82 (3.30)	0.989	0.992	0.919	0.956	0.959	0.944	0.288	0.487
42	1.06 (0.34)	60.61 (3.45)	0.993	0.994	0.936	0.969	0.967	0.953	0.314	0.536
43	1.14 (0.35)	61.39 (3.54)	0.995	0.996	0.950	0.978	0.973	0.961	0.341	0.584
44	1.22 (0.37)	62.22 (3.66)	0.996	0.997	0.960	0.984	0.979	0.968	0.369	0.630
45	1.30 (0.37)	63.04 (3.74)	0.997	0.998	0.969	0.989	0.983	0.974	0.397	0.674
46	1.39 (0.38)	63.89 (3.81)	0.998	0.998	0.976	0.992	0.986	0.979	0.431	0.720
47	1.48 (0.39)	64.79 (3.90)	0.999	0.999	0.983	0.995	0.990	0.983	0.465	0.767
48	1.57 (0.40)	65.69 (3.99)	0.999	0.999	0.986	0.997	0.992	0.986	0.499	0.800
49	1.66 (0.40)	66.57 (3.97)	0.999	0.999	0.990	0.998	0.993	0.989	0.533	0.832
50	1.77 (0.41)	67.70 (4.08)	1.000	1.000	0.993	0.999	0.995	0.991	0.574	0.866
51	1.89 (0.41)	68.88 (4.14)	1.000	1.000	0.995	0.999	0.996	0.994	0.618	0.897
52	2.05 (0.44)	70.55 (4.42)	1.000	1.000	0.997	1.000	0.998	0.996	0.674	0.928
53	2.21 (0.46)	72.13 (4.63)	1.000	1.000	0.998	1.000	0.999	0.997	0.725	0.950
54	2.51 (0.52)	75.14 (5.25)	1.000	1.000	0.999	1.000	0.999	0.999	0.806	0.975
*DP raw score*	*DP IRT* *z-score (θ) (se)*	*DP IRT t-score (se)*	***DP items***^***c***^							
			*DP1: treat patients as impersonal objects* ${P}_{4_{DP1}}$*(θ)*	*DP2: more callous toward people* ${P}_{4_{DP2}}$*(θ)*		*DP3: worry job hardening me emotionally* ${P}_{4_{DP3}}$*(θ)*	*DP4: don’t really care what happens to some patients* ${P}_{4_{DP4}}$*(θ)*		*DP5: patients blame me for some problems* ${P}_{4_{DP5}}$*(θ)*	
0	−1.52 (0.60)	34.81 (6.03)	0.004	0.000		0.002	0.003		0.059	
1	−1.13 (0.53)	38.71 (5.32)	0.007	0.000		0.006	0.005		0.087	
2	−0.78 (0.49)	42.19 (4.87)	0.014	0.001		0.015	0.009		0.122	
3	−0.57 (0.48)	44.26 (4.85)	0.020	0.003		0.026	0.012		0.148	
4	−0.41 (0.48)	45.91 (4.80)	0.026	0.005		0.039	0.015		0.171	
5	−0.29 (0.49)	47.10 (4.93)	0.032	0.008		0.054	0.018		0.190	
6	−0.15 (0.47)	48.54 (4.68)	0.041	0.014		0.076	0.022		0.214	
**7**	**0.01 (0.43)**	**50.06 (4.27)**	**0.053**	**0.026**		**0.112**	**0.029**		**0.244**	
8	0.14 (0.41)	51.39 (4.10)	0.066	0.043		0.152	0.035		0.270	
9	0.26 (0.40)	52.61 (4.01)	0.080	0.068		0.198	0.042		0.296	
10	0.38 (0.39)	53.76 (3.95)	0.097	0.105		0.253	0.050		0.324	
11	0.49 (0.39)	54.86 (3.90)	0.116	0.154		0.313	0.059		0.350	
12	0.59 (0.39)	55.94 (3.87)	0.135	0.214		0.373	0.068		0.375	
13	0.70 (0.38)	57.00 (3.85)	0.159	0.296		0.444	0.079		0.403	
14	0.80 (0.38)	58.04 (3.84)	0.184	0.385		0.511	0.092		0.428	
15	0.91 (0.38)	59.08 (3.84)	0.215	0.493		0.583	0.107		0.457	
16	1.01 (0.39)	60.11 (3.86)	0.247	0.591		0.647	0.123		0.484	
17	1.12 (0.39)	61.15 (3.90)	0.284	0.692		0.711	0.142		0.514	
18	1.22 (0.39)	62.19 (3.94)	0.322	0.770		0.762	0.162		0.540	
19	1.32 (0.40)	63.23 (3.99)	0.361	0.833		0.807	0.185		0.567	
20	1.43 (0.40)	64.29 (4.04)	0.407	0.885		0.849	0.212		0.595	
21	1.54 (0.41)	65.38 (4.10)	0.454	0.923		0.883	0.242		0.623	
22	1.65 (0.42)	66.53 (4.19)	0.503	0.949		0.910	0.274		0.650	
23	1.77 (0.43)	67.73 (4.31)	0.555	0.968		0.933	0.313		0.679	
24	1.89 (0.44)	68.94 (4.42)	0.607	0.980		0.951	0.355		0.706	
25	2.01 (0.45)	70.13 (4.49)	0.656	0.987		0.964	0.399		0.732	
26	2.14 (0.45)	71.42 (4.53)	0.706	0.992		0.974	0.448		0.758	
27	2.30 (0.46)	72.97 (4.58)	0.761	0.996		0.983	0.510		0.788	
28	2.49 (0.47)	74.93 (4.67)	0.816	0.998		0.990	0.583		0.820	
29	2.73 (0.49)	77.32 (4.94)	0.871	0.999		0.995	0.670		0.855	
30	3.05 (0.55)	80.49 (5.51)	0.923	1.000		0.998	0.770		0.893	
*PA raw score*	*PA IRT* *z-score (θ) (se)*	*PA IRT t-score (se)*	***PA items***^***d***^							
			*PA1: Easily understand how patients feel* ${P}_{4_{PA1}}$*(θ)*	*PA2: deal very effectively with problems of patients* ${P}_{4_{PA2}}$*(θ)*	*PA3: positively influencing other people’s lives through work* ${P}_{4_{PA3}}$*(θ)*	*PA4: feel very energetic* ${P}_{4_{PA4}}$*(θ)*	*PA5: can easily create relaxed atmosphere with patients* ${P}_{4_{PA5}}$*(θ)*	*PA6: exhilarated after working closely with patients* ${P}_{4_{PA6}}$*(θ)*	*PA7: accomplished many worthwhile things in job* ${P}_{4_{PA7}}\left(\theta \right)$	*PA8: deal with emotional problems very calmly* ${P}_{4_{PA8}}$*(θ)*
0	−3.91 (0.48)	10.91 (4.83)	0.217	0.083	0.002	0.013	0.019	0.002	0.001	0.110
1	−3.71 (0.45)	12.85 (4.54)	0.256	0.111	0.004	0.018	0.027	0.003	0.002	0.137
2	−3.56 (0.44)	14.36 (4.40)	0.288	0.138	0.006	0.022	0.035	0.004	0.003	0.159
3	−3.45 (0.43)	15.51 (4.33)	0.313	0.161	0.008	0.026	0.043	0.005	0.004	0.178
4	−3.34 (0.42)	16.65 (4.22)	0.340	0.187	0.010	0.030	0.052	0.006	0.005	0.199
5	−3.23 (0.42)	17.67 (4.16)	0.367	0.215	0.013	0.035	0.063	0.008	0.006	0.221
6	−3.14 (0.41)	18.64 (4.09)	0.390	0.241	0.017	0.040	0.074	0.010	0.008	0.240
7	−3.04 (0.40)	19.61 (4.01)	0.417	0.272	0.021	0.046	0.088	0.012	0.010	0.263
8	−2.95 (0.39)	20.52 (3.95)	0.441	0.302	0.027	0.052	0.102	0.014	0.013	0.285
9	−2.86 (0.39)	21.42 (3.89)	0.465	0.334	0.033	0.059	0.119	0.016	0.016	0.307
10	−2.77 (0.38)	22.28 (3.84)	0.489	0.367	0.042	0.066	0.138	0.019	0.020	0.331
11	−2.69 (0.38)	23.12 (3.79)	0.511	0.398	0.050	0.074	0.157	0.023	0.024	0.353
12	−2.61 (0.38)	23.94 (3.75)	0.533	0.430	0.061	0.082	0.178	0.026	0.029	0.375
13	−2.53 (0.37)	24.74 (3.72)	0.554	0.462	0.074	0.091	0.201	0.031	0.036	0.398
14	−2.45 (0.37)	25.52 (3.69)	0.576	0.495	0.089	0.101	0.226	0.036	0.043	0.422
15	−2.37 (0.37)	26.30 (3.66)	0.597	0.527	0.107	0.112	0.253	0.041	0.052	0.446
16	−2.29 (0.36)	27.07 (3.64)	0.618	0.560	0.129	0.124	0.283	0.048	0.063	0.470
17	−2.22 (0.36)	27.83 (3.62)	0.636	0.588	0.150	0.136	0.310	0.055	0.075	0.491
18	−2.14 (0.36)	28.58 (3.60)	0.656	0.619	0.178	0.150	0.343	0.063	0.090	0.515
19	−2.07 (0.36)	29.33 (3.59)	0.673	0.645	0.206	0.163	0.373	0.072	0.106	0.537
20	−1.99 (0.36)	30.08 (3.59)	0.692	0.675	0.241	0.180	0.409	0.083	0.126	0.561
21	−1.92 (0.36)	30.82 (3.58)	0.708	0.699	0.275	0.195	0.441	0.094	0.147	0.581
22	−1.84 (0.36)	31.57 (3.58)	0.725	0.726	0.318	0.214	0.478	0.108	0.174	0.605
23	−1.77 (0.36)	32.32 (3.58)	0.740	0.748	0.358	0.231	0.511	0.122	0.201	0.625
24	−1.69 (0.36)	33.07 (3.58)	0.757	0.772	0.406	0.253	0.548	0.139	0.236	0.647
25	−1.62 (0.36)	33.83 (3.59)	0.770	0.791	0.450	0.272	0.581	0.157	0.269	0.667
26	−1.54 (0.36)	34.60 (3.60)	0.785	0.812	0.501	0.296	0.617	0.178	0.311	0.688
27	−1.46 (0.36)	35.38 (3.61)	0.800	0.831	0.552	0.320	0.651	0.202	0.355	0.708
28	−1.38 (0.36)	36.17 (3.62)	0.813	0.849	0.601	0.346	0.685	0.229	0.403	0.728
29	−1.30 (0.36)	36.98 (3.64)	0.826	0.865	0.649	0.373	0.716	0.257	0.453	0.747
30	−1.22 (0.37)	37.80 (3.66)	0.838	0.879	0.694	0.400	0.746	0.288	0.503	0.765
31	−1.14 (0.37)	38.64 (3.68)	0.850	0.892	0.736	0.428	0.773	0.321	0.553	0.782
32	−1.05 (0.37)	39.50 (3.71)	0.862	0.906	0.778	0.460	0.801	0.361	0.609	0.800
33	−0.96 (0.37)	40.39 (3.74)	0.873	0.917	0.815	0.493	0.827	0.402	0.661	0.817
34	−0.87 (0.38)	41.30 (3.78)	0.884	0.928	0.847	0.525	0.850	0.445	0.710	0.832
35	−0.78 (0.38)	42.25 (3.82)	0.893	0.937	0.875	0.558	0.870	0.488	0.755	0.847
36	−0.68 (0.39)	43.23 (3.87)	0.903	0.946	0.900	0.593	0.890	0.537	0.799	0.862
37	−0.57 (0.39)	44.26 (3.92)	0.913	0.955	0.923	0.631	0.908	0.590	0.840	0.877
38	−0.47 (0.40)	45.33 (3.99)	0.922	0.961	0.939	0.664	0.923	0.636	0.871	0.890
39	−0.35 (0.41)	46.48 (4.07)	0.931	0.968	0.954	0.701	0.938	0.688	0.901	0.903
40	−0.23 (0.42)	47.69 (4.16)	0.939	0.973	0.966	0.736	0.949	0.736	0.925	0.915
41	−0.10 (0.43)	48.99 (4.27)	0.946	0.978	0.975	0.771	0.960	0.782	0.945	0.927
**42**	**0.04 (0.44)**	**50.38 (4.37)**	**0.953**	**0.983**	**0.983**	**0.805**	**0.969**	**0.825**	**0.961**	**0.937**
43	0.19 (0.44)	51.89 (4.42)	0.960	0.986	0.988	0.837	0.976	0.863	0.973	0.947
44	0.36 (0.45)	53.61 (4.52)	0.967	0.990	0.992	0.868	0.983	0.898	0.982	0.957
45	0.56 (0.46)	55.58 (4.62)	0.973	0.993	0.995	0.897	0.988	0.929	0.989	0.966
46	0.80 (0.47)	58.03 (4.73)	0.979	0.995	0.997	0.925	0.992	0.954	0.994	0.974
47	1.15 (0.52)	61.47 (5.21)	0.986	0.997	0.999	0.954	0.996	0.976	0.998	0.983
48	1.62 (0.61)	66.22 (6.07)	0.991	0.999	1.000	0.976	0.998	0.990	0.999	0.990

The burnout symptom burden represented by subscale scores in this table can be interpreted based on the profile of likely item endorsements (i.e., a content-referenced score interpretation) as well as how far above or below the scores are relative to the mean score in a 2014 reference population of US physicians (i.e., a norm-referenced score interpretation). ^a^ A raw score on each subscale refers to the total (or sum) score on each subscale. Bolded rows correspond to IRT scores and response profiles that are at or closest to the mean for each subscale. ^b^ Items EE4 and EE8 were combined into one item (EE4EE8) to meet local independence assumptions. The probabilities shown for the combined EE4EE8 item represent the probability of a physician endorsing that working with people puts too much stress on him/her and/or is a real strain at a frequency of once a week or more at a particular score (i.e., a score of at least 8 on the combined EE4EE8 item). Higher scores on the EE subscale indicate more emotional exhaustion (and a higher burnout symptom burden). An EE item with > 0.50 probability of endorsement indicates a physician is likely to endorse feeling that particular EE symptom at a frequency of once a week or more at a particular score. ^c^ Higher scores on the DP subscale indicate more depersonalization (and a higher burnout symptom burden). A DP item with > 0.50 probability of endorsement indicates a physician is likely to endorse feeling that particular DP symptom at a frequency of once a week or more at a particular score. ^d^ Higher scores on the PA subscale indicate more personal accomplishment (and a lower symptom burden); whereas, lower scores on the PA subscale indicate a lower sense of personal accomplishment (and higher burnout symptom burden). A PA item with < 0.50 probability of endorsement indicates a physician is unlikely to endorse feeling that particular PA indicator at a frequency of once a week or more at a particular score

#### Depersonalization subscale

A physician scoring approximately at the mean (raw score of 7) on the DP subscale is unlikely to endorse feeling any depersonalization symptoms (DP1-DP5) once weekly or more. Physicians are also unlikely to endorse any depersonalization symptoms weekly or more at the commonly used raw score cut-point of 10, which represents a z-score that is 0.38 SDs above the mean DP level of US physicians. Endorsing feeling worried that work is hardening you emotionally (DP3), more callous toward people (DP2), patients blame you (DP5), that you treat patients as impersonal objects (DP1), and that you don’t care what happens to some patients (DP4) once weekly or more is likely among physicians with z-scores > 0.78, > 0.92, > 1.07, > 1.64, and > 2.27 above the mean, respectively.

#### Personal accomplishment subscale

A physician scoring approximately at the mean (raw score of 42) on the PA subscale is likely to endorse all items (PA1-PA8) at a frequency of once weekly or more. The commonly used raw score cut-point of 33 represents a z-score that is 0.96 SDs below the mean PA level of US physicians. A physician with this score would be likely to endorse feeling he/she: can easily understand how patients feel (PA1); deals very effectively with patient problems (PA2); positively influences other people’s lives through work (PA3); can easily create a relaxed atmosphere with patients (PA5); has accomplished many worthwhile things at work (PA7); deals with emotional work problems very calmly (PA8). A physician with this score would be unlikely, however, to endorse feeling very energetic (PA4) or exhilarated after working closely with patients (PA6) weekly or more, representing several burnout symptoms. Additional symptoms of low PA are likely among physicians with z-scores less than − 1.22 SDs below the mean.

### Precision bandwidth

**Fig. 1:**
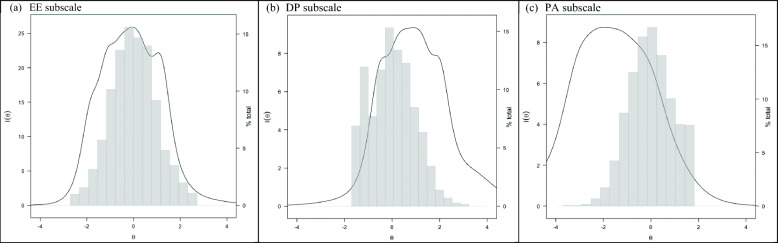
Emotional Exhaustion (EE), Depersonalization (DP), and Personal Accomplishment (PA) Subscale Test Information Functions (TIF) Relative to US Physician Sample Score Distribution ^a^.^*a*^*(I*∣*θ) of 3.33, 10, and 20 is approximately equal to 0.70, 0.90, and 0.95 reliability, respectively. θ refers to the underlying metric for each MBI subscale and corresponds with IRT z-scores.* US physicians’ z-scores are distributed from − 2.51 to 2.51, − 1.52 to 3.05, and − 3.51 to 1.62, on the EE, DP, and PA subscales, respectively

Figure 1 presents TIFs plotted against each subscale’s sample score distribution. Of the score ranges in which US physicians are distributed, the EE, DP, and PA subscales have adequate reliability for group-level measurement at respective z-scores of − 2.51 to 2.34, − 1.09 to 2.71, and − 3.51 to 0.97. Thus, 96.6%, 83.2%, 87.3% of the respective EE, DP, and PA sample score ranges can be assessed with ≥ 0.70 reliability. The EE and DP subscales do not possess adequate reliability to assess levels of EE and DP > 2.34 and > 2.71 SDs above the mean, respectively, at the highest ends of the EE and DP metrics where a physician is likely to report experiencing all EE and DP symptoms weekly or more. The DP and PA subscales do not possess adequate reliability to assess low DP levels less than 1.09 SDs below the mean and high PA levels > 0.97 SDs above the mean, corresponding to nearly no burnout symptoms. Reliability of the EE, DP, and PA scales peaked at 0.96, 0.89, and 0.89 between z-scores of − 1.19 to 0.59, 0.14 to 1.37, − 2.23 to − 1.60, respectively. Only the EE scale showed adequate reliability for individual-level measurement (from z-scores of − 2.18 to 1.76).

## Discussion

The MBI has informed much of the current US health policy discourse surrounding the physician burnout crisis and continues to be the most widely used outcome assessment to monitor physician burnout prevalence at organizational and national levels [4–6, 8, 9, 23]. However, to our knowledge, no studies have used IRT to improve what is known about its psychometric properties in a national sample of physicians. In this study, we used IRT to better understand the meaning and precision of MBI subscale scores in US physicians. After calibrating each MBI subscale, we described the burnout symptom severity represented by each subscale item; created response profiles describing the probability that a US physician endorses each item at a frequency of once weekly or more across standardized, IRT-based subscale scores; and mapped IRT-based subscale scores to raw MBI subscale scores. As an example of their utility, we used the crosswalks and response profiles to interpret the meaning of mean scores and commonly used cut-points for defining dichotomous EE, DP, and PA outcomes. These crosswalks can also be used to compare groups’ (and for the EE subscale, individuals’) scores on each metric relative to the average level of each construct in a US physician reference population.

This analysis revealed several important findings regarding the burnout symptom burden experienced by the average US physician and represented by commonly used cut-points. The average US physician is likely to experience several EE symptoms once weekly or more, including feeling emotionally drained, used up, frustrated, and working too hard due to work; is unlikely to experience any symptoms of DP once weekly or more; and is likely to experience all indicators of PA once weekly or more. At respective EE, DP, and PA cut-points of 27, 10, and 33, a physician is likely to endorse the same EE symptoms that are experienced by a physician with a mean score and is unlikely to report feeling burned out from work once weekly or more; is unlikely to experience any DP symptoms once weekly or more (or even “a few times a month” or more); and is likely to experience most indicators of PA (including feeling accomplished) once weekly or more. If a physician’s endorsement of particular symptoms on each subscale is central to the definitions of dichotomous EE, DP, and PA outcomes, then our response profiles can be used to define the raw score cut-points at which physicians are likely report a particular EE, DP, and low PA burden. For example, if feeling “burned out from work”, feeling ≥ 1 symptom of DP, and not feeling professionally accomplished at least once weekly are central to the definitions of dichotomous EE, DP, and PA outcomes, respectively, then our findings suggest that raw score cut-points of ≥ 31, ≥ 14, and ≤ 29 should be used on respective EE, DP, and PA subscales. These cut-points correspond with the score at which a physician would have > 50% chance of endorsing feeling burned out and ≥ 1 symptom of DP and < 50% chance of endorsing feeling accomplished at work once weekly or more. These cut-points also correspond with EE, DP, and PA levels that are 0.27 SDs above, 0.78 SDs above, and − 1.22 SDs below the mean of US physicians, respectively. Importantly, using a definition of high scores on EE and/or DP subscales to define burnout, use of these content-referenced cut-points would lower the national prevalence of physician burnout from 54.4% to approximately 43.3% (2709/6474) in 2014 [4, 5].

Our analyses of the MBI’s precision bandwidths demonstrated that each subscale assesses the majority of physicians’ scores with ≥ 0.70 reliability. However, the EE and DP subscales lack adequate precision to assess the scores of physicians reporting the very highest EE and DP levels on each metric. Analysis of the PA scale also revealed that this scale is most precise at assessing below average levels of PA (arguably where the precision is most important given low PA is a symptom of burnout) and lacks precision at assessing above average levels of PA. Further, while researchers have stated that the MBI can be used for individual-level outcome measurement [2, 24], only the EE subscale showed adequate reliability for individual-level measurement. These findings highlight that each metric does not measure all physicians’ scores with equal precision—outside the score range possessing ≥ 0.70 and ≥ 0.90 reliability, these scales have inadequate precision to assess between-group and within-individual differences, respectively. Adding items to each subscale could improve their reliability.

### Strengths and limitations

This is the first study to our knowledge to calibrate the MBI in a national sample of US physicians and create IRT-based response profiles mapped to raw scores. The strength of this study is that it allows investigators to classify physicians’ scores into discrete burnout outcome groups relative to 1) whether their score has met or exceeded a particular symptom burden represented by the items and 2) relative to the mean score of a US physician reference sample. This is particularly important in the absence of a gold-standard criterion for burnout. It is also important given the original cut-points for defining dichotomous outcomes on each subscale (examined herein) were selected by identifying the score corresponding with the third tercile in a large occupational sample [25]. As the scale developers and others have noted, a distributional approach such as this alone can result in somewhat arbitrary cut-points [24, 25]. The use of content-referenced score interpretations as a complement to the norm-referenced interpretations, as made possible through this study, addresses this shortcoming.

This study has several limitations. The burnout symptoms assessed by the MBI are continuous constructs, and it is important to treat scores as such where possible. Notwithstanding, its use in research to classify physicians into burned out versus non-burned out groups continues to influence healthcare policy and practice [6, 26]. Therefore, identifying the symptom burden associated with various cut-points has value. This study aims not to define new cut-points but instead to elucidate the meaning of the cut-points used to define physician burnout outcomes on MBI subscales, such that when reports state “X%” of physicians are “burned out” we have a better understanding (probabilistically) of what symptom burden level that means.

The selection of appropriate cut-points is a multi-attribute decision that depends critically on factors such as the intended purpose of assessment, the profile of burnout symptoms that are most probable at the cut-points, and consensus among investigators regarding what symptom burden matters for the purpose(s) of the assessment. This includes answering questions such as: which symptoms and symptom frequencies define burnout on each subscale; and what response probability criterion should be used to define whether a physician is likely or unlikely to report the burnout symptom? Our response profiles indicate the probability of item endorsements at a frequency of *once weekly or more* based on its prior use to define burnout in national studies [5, 15, 16], but it may be that a different symptom frequency is of interest. In this case, investigators can use the item parameter estimates (Table 2) to identify probable responses at different frequencies (see also Supplemental Appendix 4 for plotted cumulative probability curves describing the probability of a physician endorsing each subscale item at a frequency of *a few times a month or more across* IRT z-scores). Further, we use a response probability criterion of > 0.50 to define whether a physician is likely to endorse each item; however, it may be that a higher probability criterion (e.g., ≥ 0.67) is desired.

Definitions of what symptom burden matters should also consider relationship of a particular cut-point with external criterions. That is, what is the sensitivity and specificity of a particular cut-point with respect to important physician health and performance outcomes? To our knowledge, this has yet to be evaluated. Cut-points derived solely from content- and norm-referenced approaches may not be the cut-points at which sensitivity and specificity are maximized for a particular outcome. The optimal cut-point should be selected based on an evaluation of the costs and benefits of decisions resulting from its use to classify physicians into outcome groups (a property of context, not the subscales themselves) [27, 28]. For example, the costs and benefits of particular subscale cut-points for defining national physician burnout prevalence may differ substantially from those associated with identifying which physicians should receive an intervention. While cut-points may vary depending on context, there is a need for consistency in the cut-points used across studies when the purpose of assessment is estimating burnout prevalence [8]. Our findings can be used to inform consensus standards for defining outcome categories (e.g., burned out vs. not burned out; low, moderate, high symptoms) on each subscale for this purpose. However, this study does not address which subscales matter in the definition (e.g., EE and/or DP versus EE, DP, and PA, etc.) [29], which has also contributed to wide variation in prevalence estimates [8].

When using our crosswalk to interpret an individual’s/group’s score relative to its distance from the mean, it should be noted that comparisons will be relative to the mean EE, DP, and PA levels reported in this sample. While early and late responder analyses by Shanafelt et al. support the demographic representativeness of the sample [4], it is possible that the mean EE, DP, and PA levels in this calibration sample are not representative of those in the population. Findings from this study also cannot be assumed to generalize to other non-physician populations (e.g., nurses). That is, it cannot be assumed that the symptom burden represented by cut-points in this study have the same meaning in a non-physician sample without further research. Further research would be needed to place item responses from both groups onto the same metric and determine items function invariantly across physician and non-physician workers before raw scores can be assumed to represent the same symptom burden across groups.

It should be noted that the precision of each MBI subscale as implied by the crosswalks (Table 3) differs slightly from the precision of each metric reported by each TIF (Fig. 1) due to differences in estimating standard error (standard deviation of posterior distribution and square root of inverse Fisher expected information value, respectively). The use of each crosswalk requires complete responses on each MBI subscale. Finally, in the original study, item DP2 was slightly revised from the original MBI item (whereby “since I took this job” was removed from the original item: “I’ve become more callous toward people since I took this job”).

## Conclusions

We produced a crosswalk mapping raw MBI subscale scores to IRT-based, standardized scores and response profiles calibrated in a US physician sample. Our results can be used in research and practice to better understand the meaning and precision of MBI scores in US physicians and compare individual/group MBI scores against a reference population of US physicians. Our response profiles underscore that the choice of cut-points for defining categorical MBI subscale outcomes matters. Different scores have different meanings with respect to the burnout symptom burden they represent, and prevalence estimates will be directly influenced by which cut-point is chosen. Our findings can be used better inform the selection of appropriate cut-points for defining categorical physician burnout outcomes on each MBI subscale.

## Supplementary information

**Additional file 1.**

## Data Availability

This study was a re-analysis of existing data obtained upon request from the author of the original study (10.1016/j.mayocp.2015.08.023).

## Abbreviations

DP: Depersonalization
EAP: Expected a posteriori
EE: Emotional exhaustion
GRM: Graded response model
IRT: Item response theory
MBI: Maslach Burnout Inventory-Human Services Survey for Medical Personnel
PA: Personal accomplishment
PROMIS: Patient-Reported Outcome Measurement Information System
SE: Standard error
SD: Standard deviation
TIF: Test information function
US: United States

## References

[1] Maslach C, Jackson SE (1981). The measurement of experienced burnout. Journal of Occupational Behaviour.

[2] Maslach, C., Leiter, M. P., & Jackson, S. E. (2017). *Maslach Burnout Inventory Manual* (4th ed.). Menlo Park: Mind Garden, Inc.

[3] Dzau VJ, Kirch DG, Nasca TJ (2018). To care is human — Collectively confronting the clinician-burnout crisis. The New England Journal of Medicine.

[4] Shanafelt TD, Hasan O, Dyrbye LN, Sinsky C, Satele D, Sloan J, West CP (2015). Changes in burnout and satisfaction with work-life balance in physicians and the general US working population between 2011 and 2014. Mayo Clinic Proceedings.

[5] Shanafelt TD, West CP, Sinsky C, Trockel M, Tutty M, Satele DV, Carlasare LE, Dyrbye LN (2019). Changes in burnout and satisfaction with work-life integration in physicians and the general US working population between 2011 and 2017. Mayo Clinic Proceedings.

[6] Jha AK, Ilif AR, Chaoui AA (2019). A crisis in health care: A call to action on physician burnout.

[7] National Academies of Sciences, Engineering, and Medicine. (2018). *Graduate medical education outcomes and metrics: Proceedings of a workshop*. Washington, DC: The National Academies Press. 29897701

[8] Rotenstein LS, Torre M, Ramos MA, Rosales RC, Guille C, Sen S, Mata DA (2018). Prevalence of burnout among physicians: A systematic review. Journal of the American Medical Association.

[9] Brady KJS, Kazis LE, Sheldrick RC, Ni P, Trockel MT (2019). Selecting physician well-being measures to assess health system performance and screen for distress: Conceptual and methodological considerations. Current Problems in Pediatric and Adolescent Health Care.

[10] Reise SP, Haviland MG (2005). Item response theory and the measurement of clinical change. Journal of Personality Assessment.

[11] Cook KF, Victorson DE, Cella D, Schalet BD, Miller D (2015). Creating meaningful cut-scores for Neuro-QOL measures of fatigue, physical functioning, and sleep disturbance using standard setting with patients and providers. Quality of Life Research.

[12] HealthMeasures (2013). PROMIS instrument development and scientific standards version 2.0.

[13] Samejima F (1969). Estimation of latent ability using a response pattern of graded scores.

[14] De Ayala, R. J. (2013). *The theory and practice of item response theory*. New York: Guilford publications.

[15] West CP, Dyrbye LN, Satele DV, Sloan JA, Shanafelt TD (2012). Concurrent validity of single-item measures of emotional exhaustion and depersonalization in burnout assessment. Journal of General Internal Medicine.

[16] Shanafelt TD, Sinsky C, Dyrbye LN, Trockel M, West CP (2019). Burnout among physicians compared with individuals with a professional or doctoral degree in a field outside of medicine. Mayo Clinic Proceedings.

[17] Thissen D, Pommerich M, Billeaud K, Williams VS (1995). Item response theory for scores on tests including polytomous items with ordered responses. Applied Psychological Measurement.

[18] Revelle W (2018). psych: Procedures for personality and psychological research.

[19] Core Team R (2018). R: A language and environment for statistical computing.

[20] Rosseel Y (2012). Lavaan: An R package for structural equation modeling. Journal of Statistical Software.

[21] Chalmers P (2012). Mirt: A multidimensional item response theory package for the R environment. Journal of Statistical Software.

[22] Maydeu-Olivares, A. (2014). Evaluating the fit of IRT models. In S. P. Reise & D. A. Revicki (Eds.), *Handbook of item response theory modeling* (pp. 129–145). New York: Routledge.

[23] National Academy of Medicine (2018). Validated instruments to assess work-related dimensions of well-being.

[24] Schaufeli WB, Bakker AB, Hoogduin K, Schaap C, Kladler A (2001). On the clinical validity of the Maslach burnout inventory and the burnout measure. Psychology & Health.

[25] Mind Garden, Inc (2018). The problem with cutoffs for the Maslach Burnout Inventory.

[26] Health Resources & Services Administration (2017). Advisory Committee on Training in Primary Care Medicine and Dentistry (ACTPCMD) meeting minutes: March 6–7, 2017.

[27] Sheldrick RC, Garfinkel D (2017). Is a positive developmental-behavioral screening score sufficient to justify referral? A review of evidence and theory. Academic Pediatrics.

[28] Sheldrick RC, Benneyan JC, Kiss IG, Briggs-Gowan MJ, Copeland W, Carter AS (2015). Thresholds and accuracy in screening tools for early detection of psychopathology. Journal of Child Psychology and Psychiatry.

[29] Eckleberry-Hunt, J., Kirkpatrick, H., & Barbera, T. (2017). The problems with burnout research. *Academic Medicine*. 10.1097/acm.0000000000001890. 10.1097/ACM.0000000000001890 28817432

