# Supplementary material for: Describing the emotional exhaustion, depersonalization, and low personal accomplishment symptoms associated with Maslach Burnout Inventory subscale scores in US physicians: an item response theory analysis
Source: J Patient Rep Outcomes. 2020 Jun 1;4:42. doi: 10.1186/s41687-020-00204-x (PMC7266903; doi:10.1186/s41687-020-00204-x)
Supplement: Supplementary file 1 — Additional file 1. [file 41687_2020_204_MOESM1_ESM.pdf]

Supplemental Appendices:
Describing the emotional exhaustion, depersonalization, and low personal accomplishment symptoms associated with Maslach Burnout Inventory subscale scores in US physicians: an item response theory analysis

Supplemental Appendix 1: Methods of Assessing IRT Model Assumptions

1.1 Unidimensionality Assessment

We assessed the unidimensionality assumption using unidimensional and bifactor exploratory and confirmatory factor analyses (CFA). Use of the bifactor model in dimensionality assessments preceding IRT calibration is an important diagnostic tool for evaluating the degree to which items are *essentially* unidimensional for a unidimensional IRT model.[1-3] We used bifactor exploratory factor analyses (EFAs) and bifactor CFAs first to evaluate the feasibility of fitting a unidimensional IRT model to a composite burnout measure (i.e., all 22 MBI items or, separately, all 14 EE and DP items). This was performed because, to the extent that the burnout items under investigation are explained by one general underlying construct, the scaling of substantively broad (versus narrow) burnout constructs may be more meaningful and actionable to healthcare leaders (and reduce inconsistencies in determining categorical burnout outcomes on the MBI).[1,4] Essential unidimensionality is defined by the existence of a major latent dimension that accounts for common variance among all the items in a scale, despite the presence of other minor, nuisance dimensions (e.g., those caused by clusters of items with similar item content).[5] When items are found to be essentially unidimensional, the IRT assumption of unidimensionality is tenable if imposing a unidimensional latent structure to the data do not significantly bias item parameter estimates.[3,6-8] To examine the extent to which scaling each subscale in a unidimensional IRT model would distort item parameter estimates due to unmodeled multidimensionality, we examined the relative bias in factor loading estimates from the unidimensional CFA model compared to the factor loadings estimates from the bifactor CFA of the same items. Relative parameter bias was calculated for each item as the absolute difference between the item's unidimensional and bifactor general factor loadings divided by the item's general factor loading.[6] We considered relative parameter bias of less than 15% acceptable and as evidence that a composite scale was unidimensional enough to justify the scaling of the items in a

unidimensional IRT model.[6,9] Conversely, we considered relative parameter bias of ≥15% as demonstrative that unmodeled multidimensionality among composite scale items was distorting item parameter estimates.[6]

If a composite scale solution was too multidimensional for unidimensional IRT (determined using the methods/criteria above), we estimated unidimensional CFAs on subscale items separately. Exploratory and confirmatory bifactor analyses were subsequently conducted if subscale items showed inadequate fit to a unidimensional CFA model. In this case, exploratory bifactor analyses were first conducted to evaluate excess correlation among subsets of subscale items. Once specific factors were identified in an exploratory bifactor solution, we estimated a confirmatory bifactor model on the latent structure identified in the exploratory bifactor analysis. If the confirmatory bifactor solution fit the subscale items adequately, we examined relative parameter bias using the same methods described above.

Exploratory bifactor models were estimated using maximum likelihood and polychoric correlation matrices in the R *psych* package.[10] Bifactor and unidimensional CFA models were estimated using mean- and variance-adjusted weighted least squares (WLSMV) and polychoric correlation matrices on pairwise complete observations in the R *lavaan* package (version 0.6-3).[11] Adequate fit in the CFA models was defined as a scaled Root Mean Square Error Approximation (RMSEA) of ≤0.10, a scaled Tucker-Lewis Index (TLI) of ≥0.90, a scaled Comparative Fit Index (CFI) of ≥0.90, and a Standardized Root Mean Square Residual (SRMSR) of ≤0.08 (scaled fit indices replace the uncorrected chi-square statistic with the WLSMV robust chi-square in the formulas for the RMSEA, CFI, and TLI).[2,12-18]

1.2 Local Independence

The assumption of local independence was evaluated using Chen and Thissen (1997) local dependence (LD) X^2 statistics.[19] The Chen and Thissen (1997) LD X^2 tests for the association between pairs of items based on the marginal tables of observed and expected response frequencies for item pairs.[19] We report LD X^2 values and standardized LD X^2 statistics in the form of Cramer's V coefficients.[19,20] The Cramer's V coefficient represents the strength of the association between pairs of categorical items, where 0 = no association and 1 = complete association. We considered item pairs with a

Cramer's V coefficient of ≥0.20, representing moderate association or more,[21] to be a violation of the local independence assumption. Violations of local independence were remedied by combining the offending items into a new single item and recalibrating the IRT model. We did this by summing the response scales for the offending items (e.g., two items with response scales coded 0 to 6 would become a single item with a response scale coded from 0 to 12). This approach avoids the problem of having to remove items from an IRT calibration because of local dependence violations. The ability to retain all items from a scale in an IRT calibration was critical given the goal of the analysis, which is to evaluate the psychometric performance of an existing scale (versus developing a new scale).

1.3 Monotonicity

To evaluate monotonicity, we calculated mean rest-scores for each item's response categories (calculated for each item category as the mean of raw scores minus the score for the item under investigation for persons who endorsed the item category) and examined whether mean rest-scores were increasing as the score category increased.[2,18,22] If a response category (*m*) showed a lower mean rest-score than its lower adjacent category (*m-1*), we performed an independent samples t-test to test the null hypothesis of equal population means (two-sided, $\alpha < 0.05$).[22] We considered a significant result a violation of monotonicity.

1.4 IRT Model Calibration and Fit

In the calibration phase, we estimated the IRT item parameters for each subscale using a unidimensional, graded response model (GRM) and marginal maximum likelihood (MML) estimation.[23] We considered a SRMSR of ≤0.05 and ≤0.08 as indicative of good and adequate overall IRT model fit, respecitvely.[24]

1.5 Examining Item Fit

Item fit examines the degree to which observed and predicted response frequencies across persons are consistent for each item.[25] For each calibrated IRT model, we initially examined item fit using the generalized signed Pearson's X^2 (S-X^2) statistic for polytomous data.[26-29] The generalized S-X^2 statistic computes a chi-square-based statistic from comparisons of observed and model-predicted

response category frequencies across each raw (sum) score group for the item of interest.[29] The null hypothesis is that there are no significant differences between the observed and expected response category frequencies across levels of the raw score, indicating appropriate item fit; whereas, the alternative hypothesis is that there are significant differences. A significant $S\text{-}X^2$ is suggestive of item misfit and requires further investigation. To control for Type I error, we applied a Benjamini-Hochberg correction to the $S\text{-}X^2$ associated p-values. Item misfit was defined as $\alpha < 0.01$ after Benjamini-Hochberg correction.

Because small deviations between expected and observed response frequencies can result in a significant $S\text{-}X^2$ with large sample sizes, we performed several additional analyses. First, we calculated the $S\text{-}X^2$ statistic again based on a random subsample of 800 observations drawn from the larger calibration sample for each measure and evaluated whether misfit remained significant ($p < 0.01$ after Benjamini-Hochberg correction).[30] Second, we evaluated the magnitude of item misfit using empirical item plots. Empirical item plots plotted the expected item score across the latent (θ) distribution against observed scores for groups of approximately 400 respondents across the latent distribution (observed scores within each ability group were calculated as the mean item score among persons in the group). The presence of item misfit following all of these evaluations was criteria for item removal (and warranted recalibration of the scale post removal). In accordance with NIH PROMIS standards, a misfitting item could be retained if its content was important conceptually to the latent construct and/or if no better alternative item was available.[2]

1.6 Examining Person Fit

Person fit examines the degree to which observed and predicted responses across items (i.e., response patterns) are consistent for each person.[25] Person misfit is indicative of aberrant response patterns, which can be caused, for example, by carelessness or cognitive errors.[25,31] When person misfit occurs, the latent score estimated for the response pattern may be invalid.[32] We used the Drasgow et al. (1985) Z_h index, a standardized likelihood-based index, to examine person fit.[33] The Z_h statistic has an approximate standard normal distribution, with values <-2 indicative of person misfit.[34] We defined

significant misfit as a Z_h value $\leq$ -3, which uses a more conservative cutoff to exclude only cases with extreme misfit. Cases with significant misfit were removed from the analysis and the IRT model was recalibrated on the basis that these individuals cannot be meaningfully scaled.[25,30,35]

Supplemental Appendix 2: IRT Assumption Assessment Results

2.1 Essential Unidimensionality Assessment

Comparison of unidimensional and bifactor CFAs on all 22 items and, separately, on all 14 EE and DP items demonstrated that several items would be distorted by unmodeled multidimensionality if a unidimensional IRT model were fit to all 22 MBI items or to all 14 EE and DP items. Mean relative parameter bias in the unidimensional model with all MBI items was 27.72% (SD = 38.12%), with 12 items (EE1, EE2, EE7, DP1, DP2, PA1, PA2, PA3, PA5, PA6, PA7, PA8) demonstrating ≥15% relative parameter bias. Mean relative parameter bias in the unidimensional model with all 14 EE and DP items was 13.37 (SD = 7.8), with 6 items (EE1, EE2, EE8, DP1, DP2, DP4) demonstrating ≥15% relative parameter bias. Given this result, we subsequently assessed unidimensionality of each MBI subscale and separately. The final CFA models for each subscale are listed below. All models supported the unidimensionality (or essential unidimensionality) of each subscale.

Supplemental Appendix 2.1 Table 1. Final CFA Model Fit Indices

**Model**	**TLI**	**CFI**	**RMSEA**	**SRMSR**	**Mean relative parameter bias (SD)**
Bifactor EE model: items EE1-EE3, EE5-EE7, EE4EE8, EE9 on general factor; EE6 and EE7 on specific factor 1; EE1 and EE2 on specific factor 2	0.997	0.998	0.074	0.017	1.71 (0.91)
Bifactor DP model: items DP1-DP5 on general factor; DP2 and DP3 on specific factor 1; DP1, DP4, DP5 on specific factor 2	0.983	0.998	0.096	0.014	5.63 (4.35)
Unidimensional PA model: PA1-PA8 on factor 1	0.900	0.929	0.102	0.066	n/a

2.2 Local Independence Evaluation

Supplemental Appendix 2.2 Table 1. MBI Emotional Exhaustion LD matrix (lower triangle) and Standardized LD Statistics (Cramer's V Coefficients, upper triangle)

	mbi_ee1	mbi_ee2	mbi_ee3	mbi_ee5	mbi_ee6	mbi_ee7	mbi_ee4ee8	mbi_ee9
mbi_ee1	NA	0.11	-0.06	-0.08	-0.06	-0.08	-0.10	-0.09
mbi_ee2	431.39	NA	0.10	-0.09	-0.08	0.06	-0.10	-0.10
mbi_ee3	-152.54	360.48	NA	0.07	-0.09	-0.08	0.10	-0.07
mbi_ee5	-231.93	-320.90	166.07	NA	-0.09	-0.09	0.11	0.10
mbi_ee6	-157.31	-253.72	-277.14	-284.24	NA	0.13	-0.12	0.08
mbi_ee7	-243.64	130.20	-253.70	-283.08	648.18	NA	-0.10	-0.08
mbi_ee4ee8	-414.35	-361.55	369.52	432.76	-498.85	-375.92	NA	0.09
mbi_ee9	-307.82	-362.73	-207.62	367.91	221.11	-257.56	294.33	NA

Supplemental Appendix 2.2 Table 2. MBI Depersonalization LD matrix (lower triangle) and Standardized LD Statistics (Cramer's V Coefficients, upper triangle)

	mbi_dp1	mbi_dp2	mbi_dp3	mbi_dp4	mbi_dp5
mbi_dp1	NA	-0.09	-0.11	0.11	0.07
mbi_dp2	-344.63	NA	0.17	-0.10	-0.06
mbi_dp3	-447.67	1124.78	NA	-0.10	0.07
mbi_dp4	438.70	-410.63	-399.84	NA	0.07
mbi_dp5	180.96	-158.16	198.19	169.26	NA

Supplemental Appendix 2.2 Table 3. MBI Personal Accomplishment LD matrix (lower triangle) and Standardized LD Statistics (Cramer's V Coefficients, upper triangle)

	mbi_pa1	mbi_pa2	mbi_pa3	mbi_pa4	mbi_pa5	mbi_pa6	mbi_pa7	mbi_pa8
mbi_pa1	NA	0.08	0.06	-0.06	0.07	-0.07	-0.06	0.06
mbi_pa2	267.91	NA	0.08	-0.08	0.10	-0.09	-0.08	0.08
mbi_pa3	120.08	252.94	NA	-0.07	-0.09	-0.08	0.07	-0.07
mbi_pa4	-139.57	-233.64	-163.56	NA	-0.09	0.10	0.07	-0.07
mbi_pa5	205.51	374.95	-283.20	-270.11	NA	0.12	-0.09	0.10
mbi_pa6	-199.86	-314.57	-252.47	337.22	506.54	NA	0.13	-0.09
mbi_pa7	-121.75	-232.42	178.77	169.20	-328.62	629.99	NA	-0.08
mbi_pa8	148.81	235.84	-196.00	-185.09	340.90	-312.08	-218.69	NA

2.3 IRT Model Fit

Supplemental Appendix 2.3 Table 1. Square Root Mean Residual (SRMR) for each calibrated IRT Model

	EE model	DP model	PA model
SRMR	0.028	0.046	0.065

2.4 Item Fit

Supplemental Appendix 2.4 Table 1. MBI Emotional Exhaustion Scale S-X^2 statistics in complete sample (n = 6,264)

Item	S-X^2	S-X^2 df	S-X^2 RMSEA	S-X^2 p-value	Benjamini Hochberg corrected S-X^2 p-value
mbi_ee1	182.05	140	0.0069	**0.0097**	**0.0115**
mbi_ee2	290.39	148	0.0124	**0.0000**	**0.0000**
mbi_ee3	184.29	183	0.0011	0.4593	0.4593
mbi_ee5	203.38	159	0.0067	0.0100	0.0115
mbi_ee6	311.93	187	0.0103	**0.0000**	**0.0000**
mbi_ee7	315.04	202	0.0095	**0.0000**	**0.0000**
mbi_ee4ee8	663.95	306	0.0137	**0.0000**	**0.0000**
mbi_ee9	334.52	198	0.0105	**0.0000**	**0.0000**

**bolded Benjamini-Hochberg corrected p-values indicate significant item misfit at p <0.01.*

Supplemental Appendix 2.4 Table 2. MBI Depersonalization Scale S-X^2 statistics in complete sample (n = 6,403)

Item	S-X^2	S-X^2 df	S-X^2 RMSEA	S-X^2 p-value	Benjamini Hochberg corrected S-X^2 p-value
mbi_dp1	212.44	100	0.0133	0.0000	**0.0000**
mbi_dp2	296.34	81	0.0204	0.0000	**0.0000**
mbi_dp3	362.56	89	0.0219	0.0000	**0.0000**
mbi_dp4	274.25	103	0.0161	0.0000	**0.0000**
mbi_dp5	125.01	101	0.0061	0.0529	0.0529

**bolded Benjamini-Hochberg corrected p-values indicate significant item misfit at p <0.01.*

Supplemental Appendix 2.4 Table 3. MBI Personal Accomplishment Scale S-X^2 statistics in complete sample (n = 6,201)

Item	S-X^2	S-X^2 df	S-X^2 RMSEA	S-X^2 p-value	Benjamini Hochberg corrected S-X^2 p-value
mbi_pa1	336.55	147	0.0144	0.0000	**0.0000**
mbi_pa2	312.50	142	0.0139	0.0000	**0.0000**
mbi_pa3	158.21	128	0.0062	0.0361	0.0361
mbi_pa4	202.84	142	0.0083	0.0006	0.0007
mbi_pa5	269.94	146	0.0117	0.0000	**0.0000**
mbi_pa6	285.48	123	0.0146	0.0000	**0.0000**
mbi_pa7	198.44	119	0.0104	0.0000	**0.0000**
mbi_pa8	304.60	146	0.0132	0.0000	**0.0000**

**bolded Benjamini-Hochberg corrected p-values indicate significant item misfit at p <0.01.*

Supplemental Appendix 2.4 Table 4. MBI Emotional Exhaustion Scale S-X^2 statistics in random subsample (n = 800)

Item	S-X^2	S-X^2 df	S-X^2 RMSEA	S-X^2 p-value	Benjamini Hochberg corrected S-X^2 p-value
mbi_ee1	84.91	91	0.0000	0.6600	0.7543
mbi_ee2	100.12	91	0.0112	0.2408	0.6421
mbi_ee3	119.40	117	0.0051	0.4210	0.6799
mbi_ee5	102.98	104	0.0000	0.5100	0.6799
mbi_ee6	141.00	120	0.0148	0.0924	0.3697
mbi_ee7	133.58	134	0.0000	0.4939	0.6799
mbi_ee4ee8	232.03	186	0.0176	0.0123	0.0983
mbi_ee9	105.07	127	0.0000	0.9225	0.9225

**bolded Benjamini-Hochberg corrected p-values indicate significant item misfit at p <0.01.*

Supplemental Appendix 2.4 Table 5. MBI 5-item Depersonalization Scale S-X^2 statistics in random subsample (n = 800)

Item	S-X^2	S-X^2 df	S-X^2 RMSEA	S-X^2 p-value	Benjamini Hochberg corrected S-X^2 p-value
mbi_dp1	89.37	71	0.0180	0.0694	0.1397
mbi_dp2	68.80	57	0.0161	0.1360	0.1397
mbi_dp3	97.93	65	0.0252	0.0052	0.0259
mbi_dp4	79.57	67	0.0153	0.1397	0.1397
mbi_dp5	100.73	83	0.0164	0.0901	0.1397

**bolded Benjamini-Hochberg corrected p-values indicate significant item misfit at p <0.01.*

Supplemental Appendix 2.4 Table 6. MBI 8-item Personal Accomplishment Scale S-X^2 statistics in random subsample (n = 800)

Item	S-X^2	S-X^2 df	S-X^2 RMSEA	S-X^2 p-value	Benjamini Hochberg corrected S-X^2 p-value
mbi_pa1	102.87	68	0.0253	0.0041	0.0324
mbi_pa2	75.10	50	0.0251	0.0123	0.0494
mbi_pa3	72.74	58	0.0178	0.0921	0.1842
mbi_pa4	81.99	74	0.0116	0.2455	0.3165
mbi_pa5	78.11	66	0.0152	0.1461	0.2338
mbi_pa6	83.95	81	0.0067	0.3893	0.3893
mbi_pa7	81.65	64	0.0186	0.0676	0.1803
mbi_pa8	80.73	74	0.0107	0.2770	0.3165

**bolded Benjamini-Hochberg corrected p-values indicate significant item misfit at p <0.01.*

Supplemental Appendix 2.4 Figure 1. MBI Emotional Exhaustion Empirical Item Plots

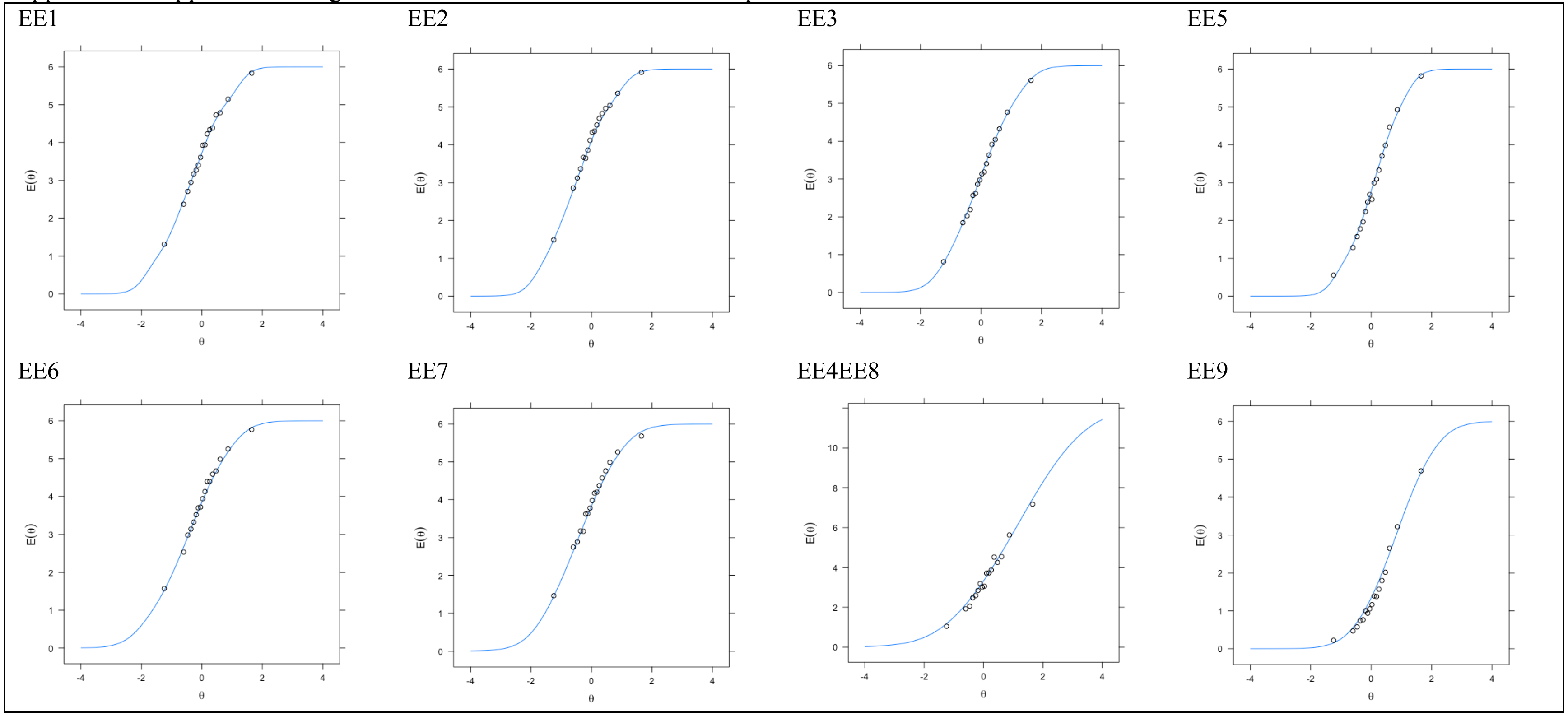

**Solid line represents the item score across the θ distribution predicted by the IRT model; dots represent observed item scores for groups of approximately 400 respondents across the latent distribution. Observed scores within each severity (traditionally called "ability") group were calculated as the mean item score among persons in the group. Higher scores indicate more emotional exhaustion.*

Supplemental Appendix 2.4 Figure 2. MBI 5-item Depersonalization Scale Empirical Item Plots

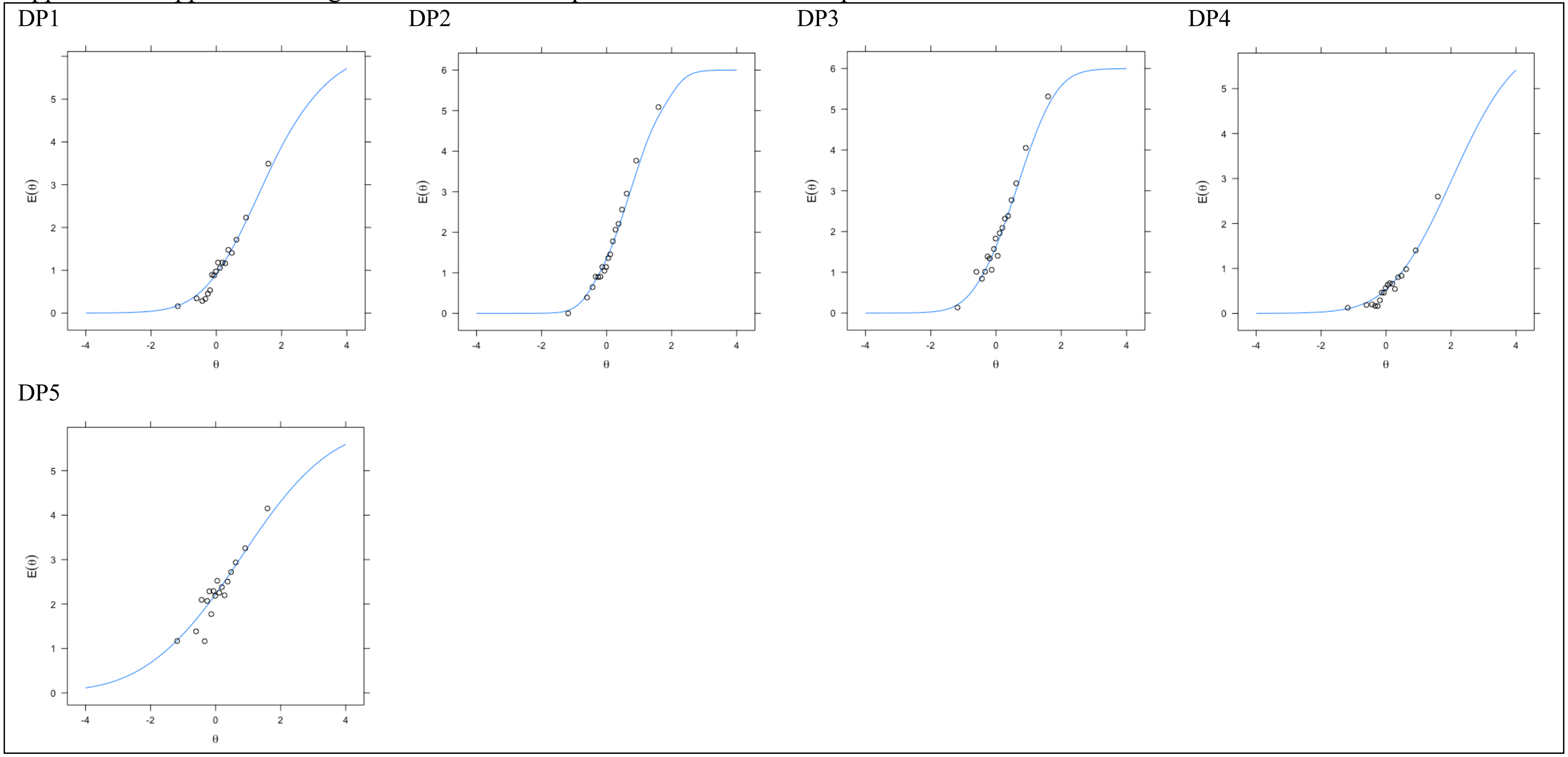

**Solid line represents the item score across the θ distribution predicted by the IRT model; dots represent observed item scores for groups of approximately 400 respondents across the latent distribution. Observed scores within each severity (traditionally called "ability") group were calculated as the mean item score among persons in the group. Higher scores indicate more depersonalization.*

Supplemental Appendix 2.4 Figure 3. MBI 8-item Personal Accomplishment Empirical Item Plots

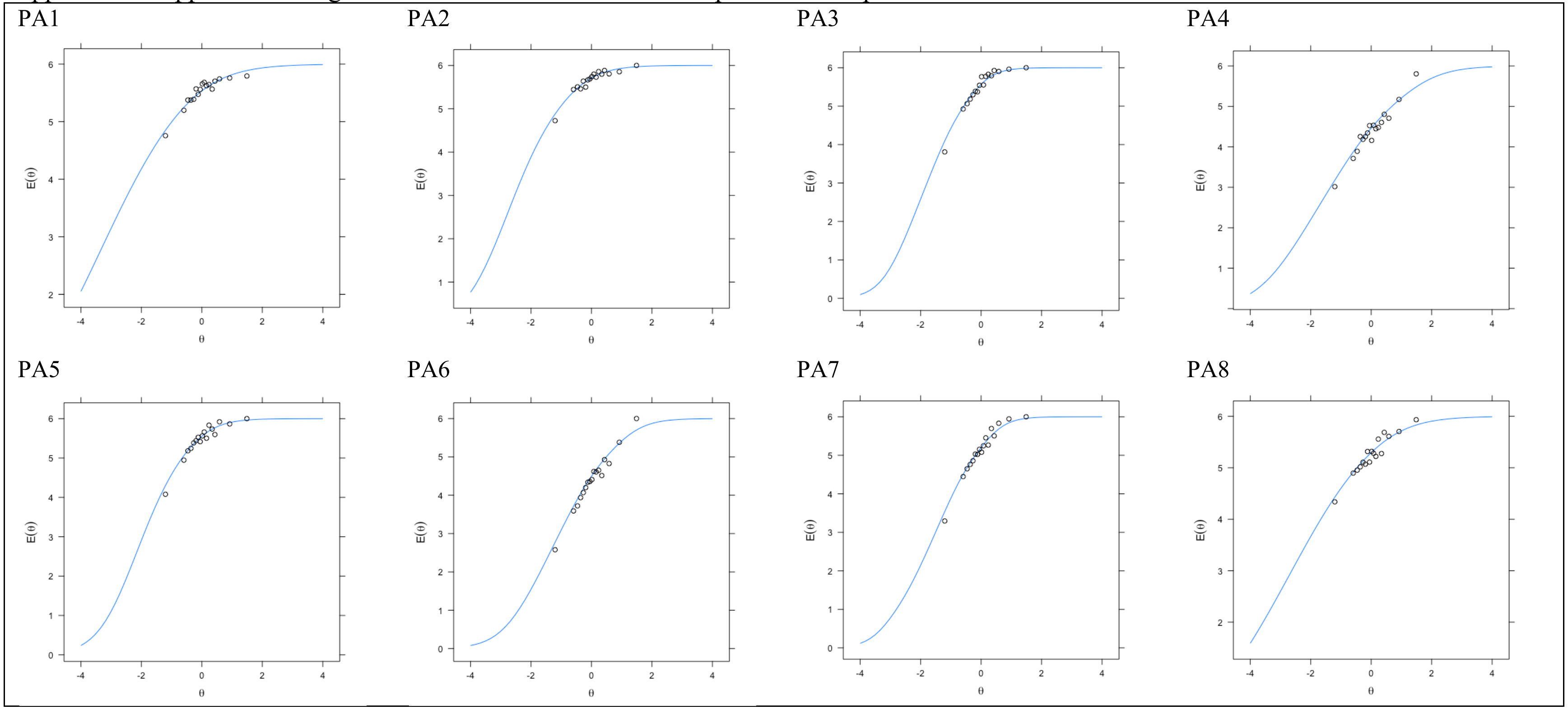

**Solid line represents the item score across the θ distribution predicted by the IRT model; dots represent observed item scores for groups of approximately 400 respondents across the latent distribution. Observed scores within each severity (traditionally called "ability") group were calculated as the mean item score among persons in the group. Higher scores indicate a higher sense of personal accomplishment.*

2.5 Person Fit

Supplemental Appendix 2.5 Figure 1. EE, DP, and PA Subscale Person Fit Z_h Distributions [a]

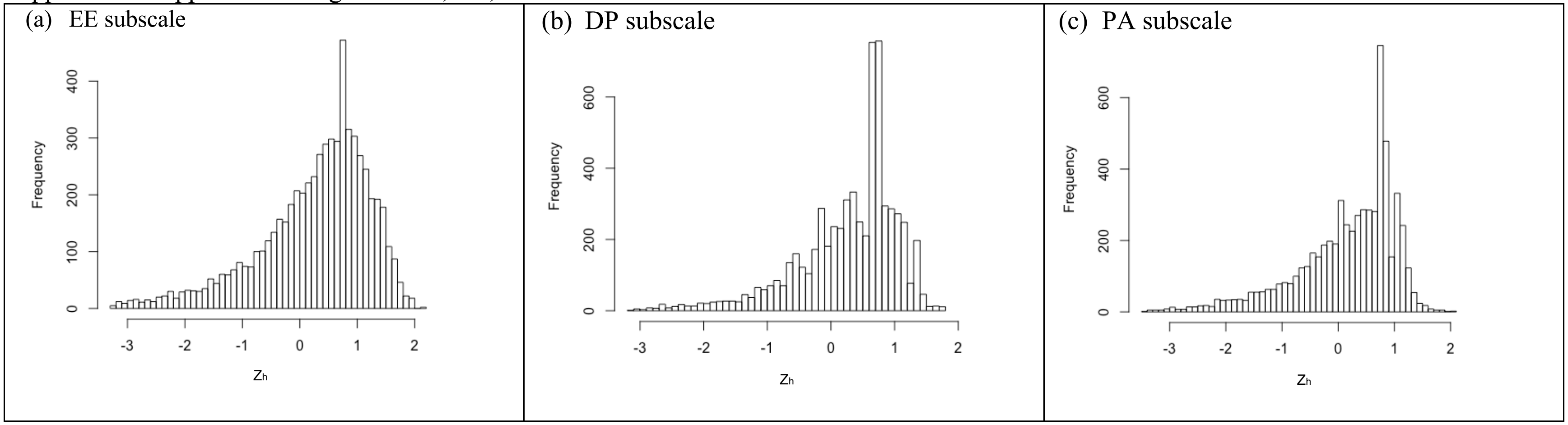

[a]n = 88, 29, and 88 physicians in the EE, DP, and PA samples with Z_h values ≤-3. These individuals were removed from the final calibrated IRT models.

2.6 Monotonicity Evaluation

Supplemental Appendix 2.6 Table 1. EE subscale Mean Rest-Scores across Item Response Categories [a]

Item	Item Response Category Mean Rest Scores						
	**Never (0)**	**A few times a year or less (1)**	**Once a month or less (2)**	**A few times a month (3)**	**Once a week (4)**	**A few times a week (5)**	**Every day (6)**
EE1	3.47	8.43	13.28	18.35	23.64	29.89	38.51
EE2	3.60	7.36	11.96	16.08	21.63	27.40	36.15
EE3	8.01	12.94	17.82	22.55	27.20	31.57	38.69
EE5	7.47	14.14	19.91	23.77	28.43	31.86	39.44
EE6	4.72	9.17	13.20	18.14	22.78	27.80	34.94
EE7	6.60	10.53	14.29	18.41	23.08	27.23	33.87
EE9	15.10	22.32	28.54	32.68	34.38	37.72	40.87

[a]*Bolded values indicate a significant Welch's two sample t-test for differences between adjacent item response category mean rest scores at alpha < 0.05. Mean rest scores for item* EE4EE8 for categories 0-12 are: 14.16, 16.89, 18.51, 22.00, 24.89, 27.01, 28.48, 30.43, 30.91, 33.13, 34.65, 36.71, 39.34.

Supplemental Appendix 2.6 Table 2. DP Subscale Mean Rest-Scores across Item Response Categories [a]

Item	Item Response Category Mean Rest-Scores						
	**Never (0)**	**A few times a year or less (1)**	**Once a month or less (2)**	**A few times a month (3)**	**Once a week (4)**	**A few times a week (5)**	**Every day (6)**
DP1	4.34	6.64	9.05	11.10	12.77	13.99	14.72
DP2	2.59	4.57	6.91	8.68	10.93	12.76	15.27
DP3	2.54	4.36	6.44	7.85	9.17	10.62	12.32
DP4	5.32	8.46	11.05	13.08	15.65	**16.69**	**13.67**
DP5	3.10	3.79	5.44	6.60	7.84	9.30	11.73

[a]*Bolded values indicate a significant Welch's two sample t-test for differences between adjacent item response category mean rest scores at alpha < 0.05.*

Supplemental Appendix 2.6 Table 3. PA subscale Mean Rest-Scores across Item Response Categories [a]

Item	Item Response Category Mean Rest-Scores						
	**Never (0)**	**A few times a year or less (1)**	**Once a month or less (2)**	**A few times a month (3)**	**Once a week (4)**	**A few times a week (5)**	**Every day (6)**
PA1	30.86	29.74	28.35	29.01	30.43	32.35	36.27
PA2	**30.28**	**24.11**	24.10	25.71	27.64	31.51	36.35
PA3	23.65	23.40	25.55	27.73	29.95	33.44	37.52
PA4	30.52	29.92	31.21	32.97	33.91	37.11	39.45
PA5	**28.43**	**24.43**	25.92	27.04	29.73	32.97	37.16
PA6	28.64	29.56	30.84	33.20	34.50	37.28	39.76
PA7	18.57	25.70	26.97	30.26	32.03	35.16	38.31
PA8	28.76	29.38	27.94	29.99	30.84	34.29	36.88

[a]*Bolded values indicate a significant Welch's two sample t-test for differences between adjacent item response category mean rest scores at alpha < 0.05.*

Supplemental Appendix 3:

Supplemental Appendix 3 Figure 1. Probability of a US physician endorsing each subscale item at a frequency of "once a week" or more across IRT z-scores [a]

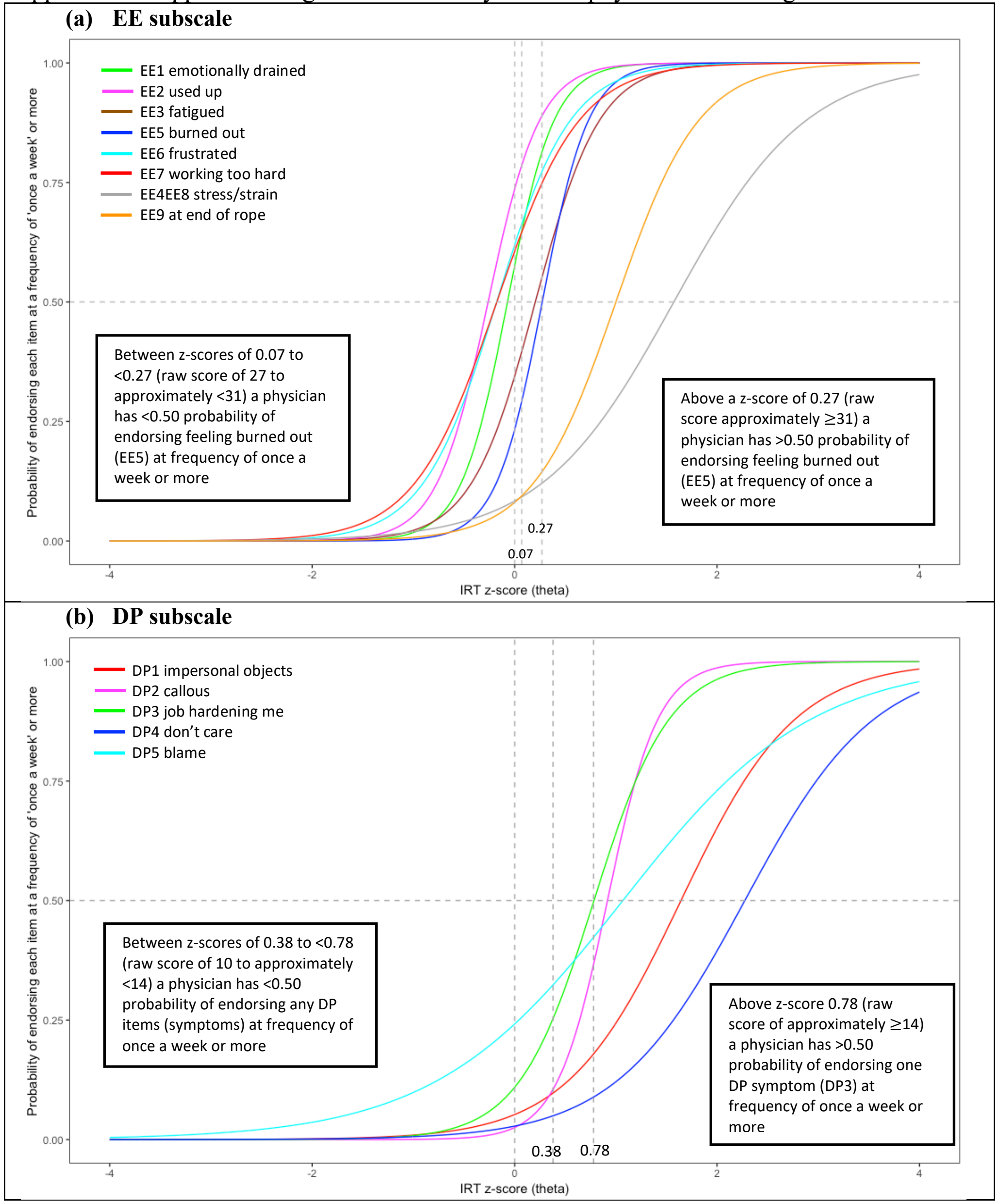

(c) PA subscale

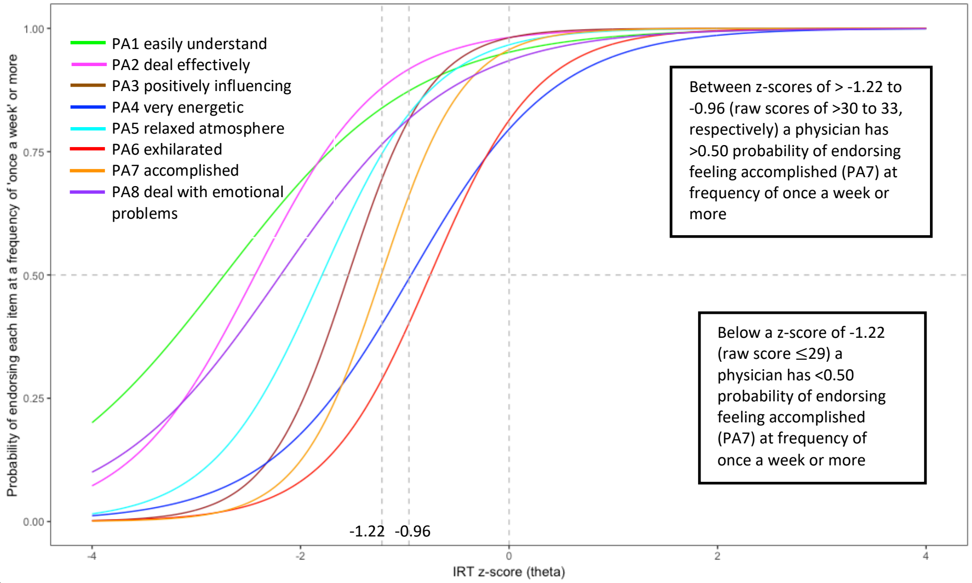

[a] Higher scores indicate higher EE, DP, and PA on each respective subscale. Higher scores on the EE and DP subscales indicate a higher burnout symptom burden. Lower scores on the PA subscale indicates a higher burnout symptom burden. EE and DP items located further to the right of the scale are items that represent a higher burnout symptom severity compared to items located further to the left of the scale. For example, at a probability of 0.50, item EE2 (left most curve in pane A) represents a below average level of EE and the least severe EE symptom (i.e., item) among all EE symptoms (i.e., items); item EE4EE8 (right most curve in pane A) represents an above average level of EE and the most severe EE symptom among all the EE symptoms; item DP3 (left most curve in pane B) represents an above average level of DP but the least severe DP symptom (i.e., item) among all DP symptoms (i.e., items), and item DP4 (right most curve in pane B) also represents an above average level of DP but the most severe DP symptom among all the DP symptoms. PA items located further to the left of the PA scale represent a higher burnout symptom severity compared to items located further to the right of the scale. For example, at a probability of 0.50, item PA1 (left most curve in pane C) represents a below average level of PA and the most severe indicator of burnout (when it is unlikely to be endorsed) among all the PA items; item PA6 (right most curve in pane C) also represents a below average level of PA but the least severe indicator of burnout (when it is unlikely to be endorsed) among all the PA items.

Option Response Functions for the EE, DP, and PA subscales

Supplemental Appendix 3 Figure 2. MBI EE Scale Option Response Functions [a]

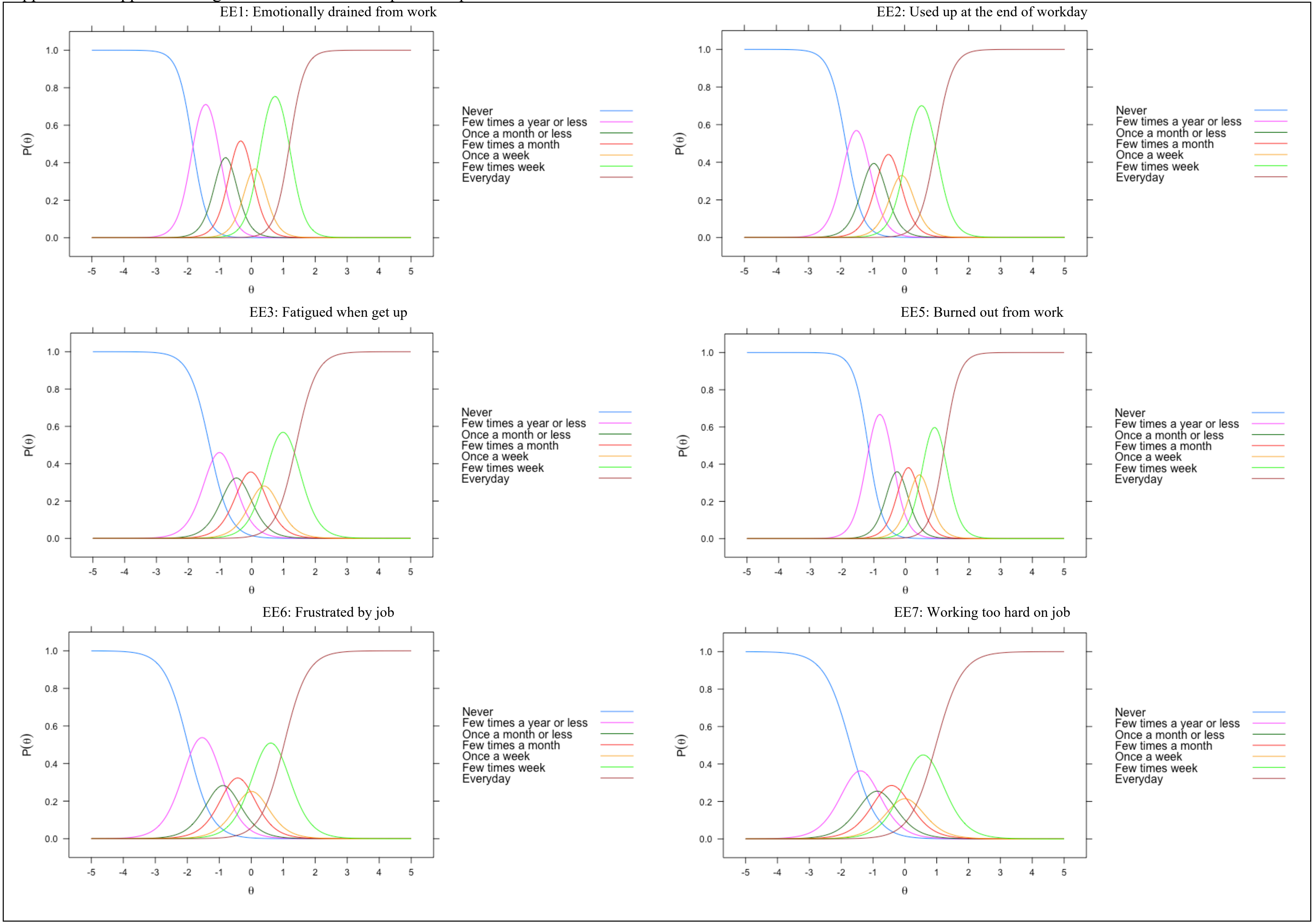

EE9: At the end of my rope

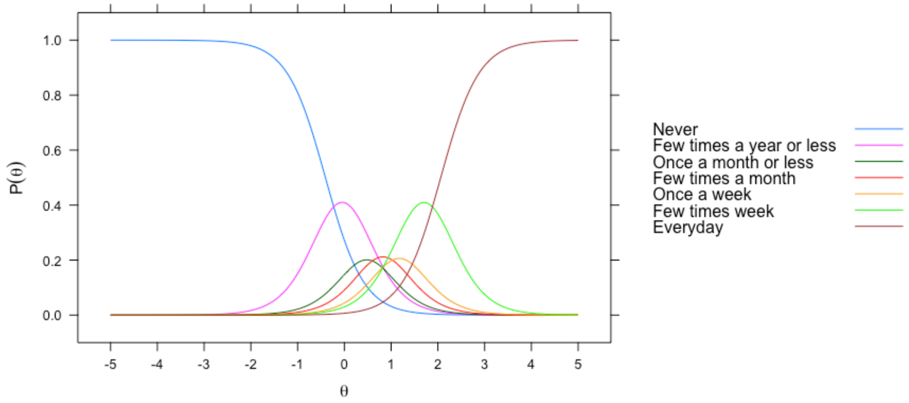

[a] Option response functions describe the probability of a random physician responding in a particular response category given a particular IRT z-score (θ). The x-axis (θ) corresponds with physicians' IRT z-scores. These ORFs can be used as an alternative to the response profiles to predict what particular response category a physician is likely to score in on a particular item given a particular score (whereas, for a particular item, the response profiles predict whether a physician is likely to endorse the response category of "once a week" or a higher response category given a particular score).

Supplemental Appendix 3 Figure 2. Depersonalization Subscale Option Response Functions [a]

DP1: Treat patients as impersonal objects

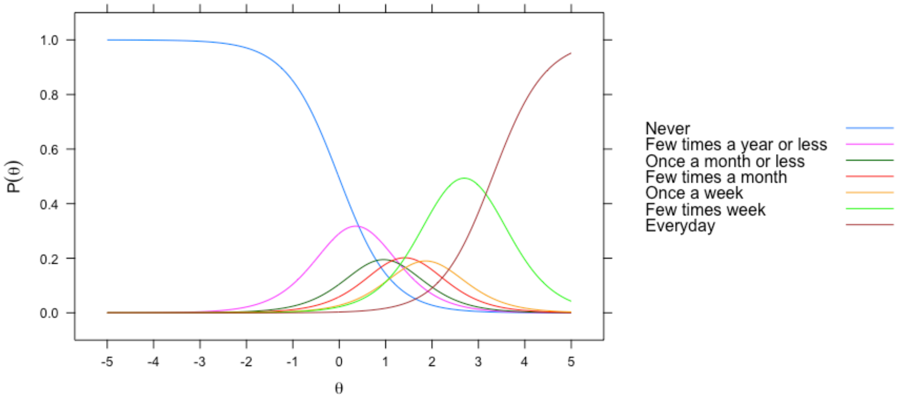

DP2: More callous toward people

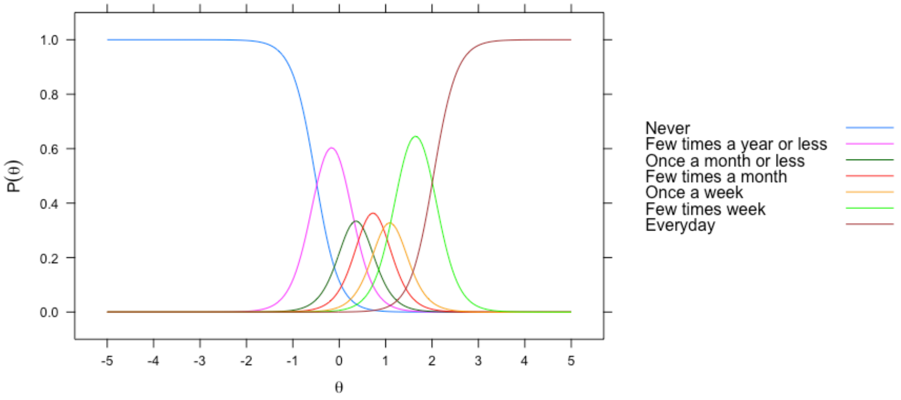

DP3: Worry job hardening me emotionally

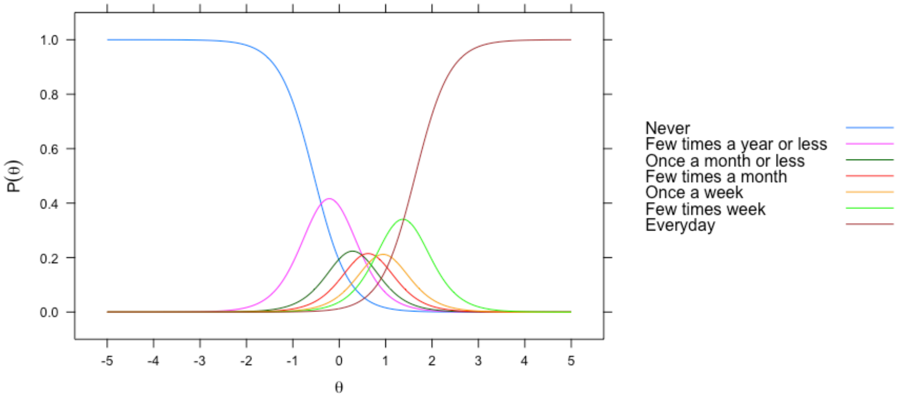

DP4: Don’t really care what happens to some patients

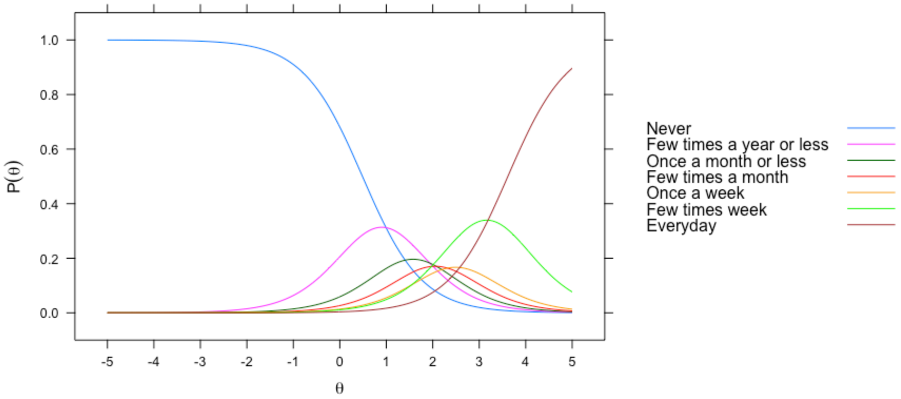

DP5: Patients blame me for some problems

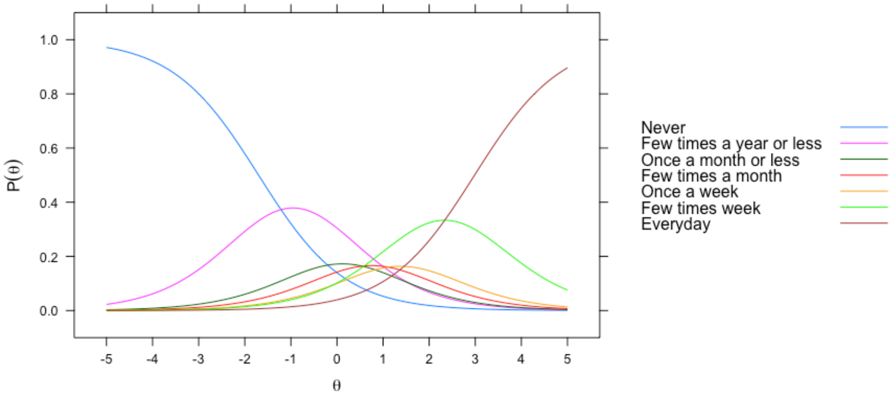

[a] Option response functions (ORFs) describe the probability of a random physician responding in a particular response category given a particular IRT z-score (θ). The x-axis (θ) corresponds with physicians’ IRT z-scores. These ORFs can be used as an alternative to the response profiles to predict what particular response category a physician is likely to score in on a particular item given a particular score (whereas, for a particular item, the response profiles predict whether a physician is likely to endorse the response category of “once a week” or a higher response category given a particular score).

Supplemental Appendix 3 Figure 3. Personal Accomplishment Subscale Option Response Functions [a]

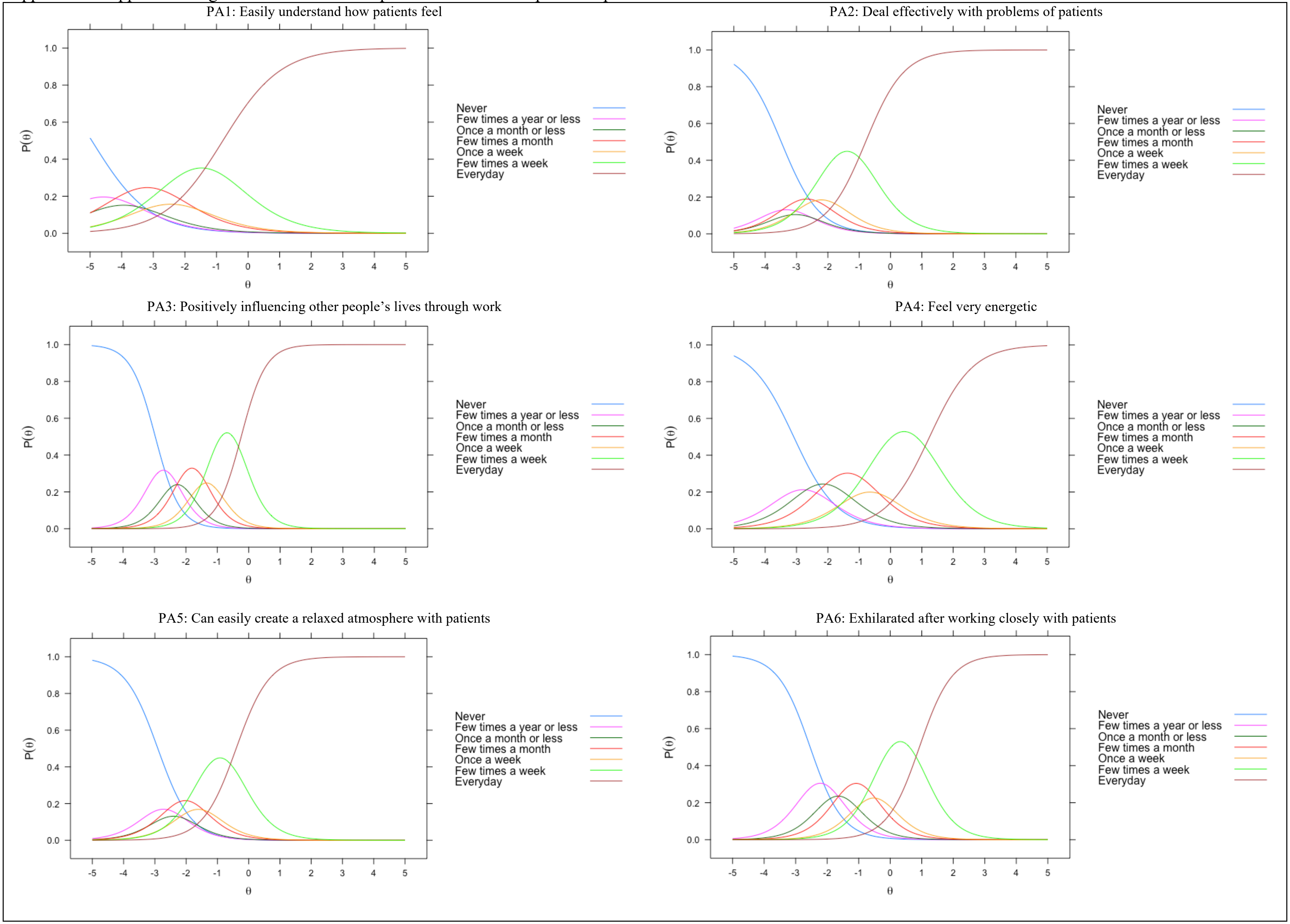

PA7: Accomplished worthwhile things in job

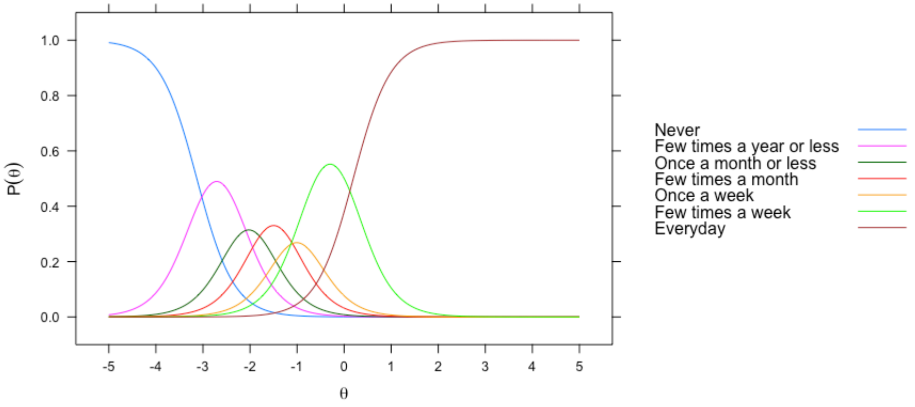

PA8: Deal with emotional problems very calmly

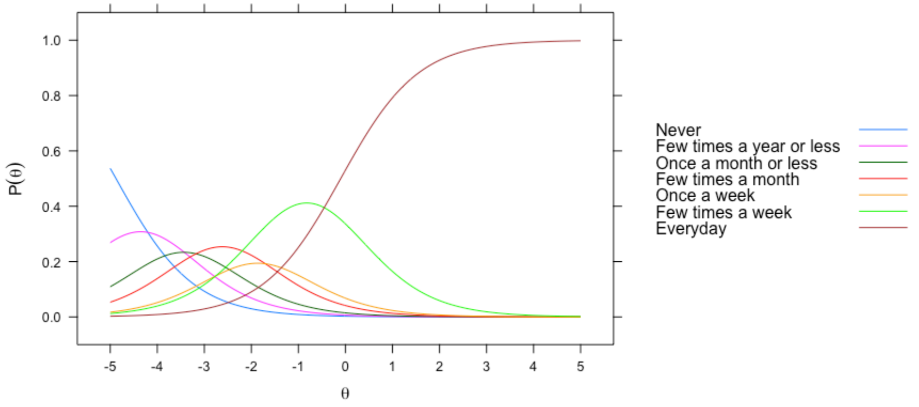

[a] Option response functions describe the probability of a random physician responding in a particular response category given a particular IRT z-score (θ). The x-axis (θ) corresponds with physicians' IRT z-scores.These ORFs can be used as an alternative to the response profiles to predict what particular response category a physician is likely to score in on a particular item given a particular score (whereas, for a particular item, the response profiles predict whether a physician is likely to endorse the response category of "once a week" or a higher response category given a particular score)

Supplemental Appendix 4:

Supplemental Appendix 4 Figure 1. Probability of a US physician endorsing each subscale item at a frequency of "a few times a month" or more across IRT z-scores [a]

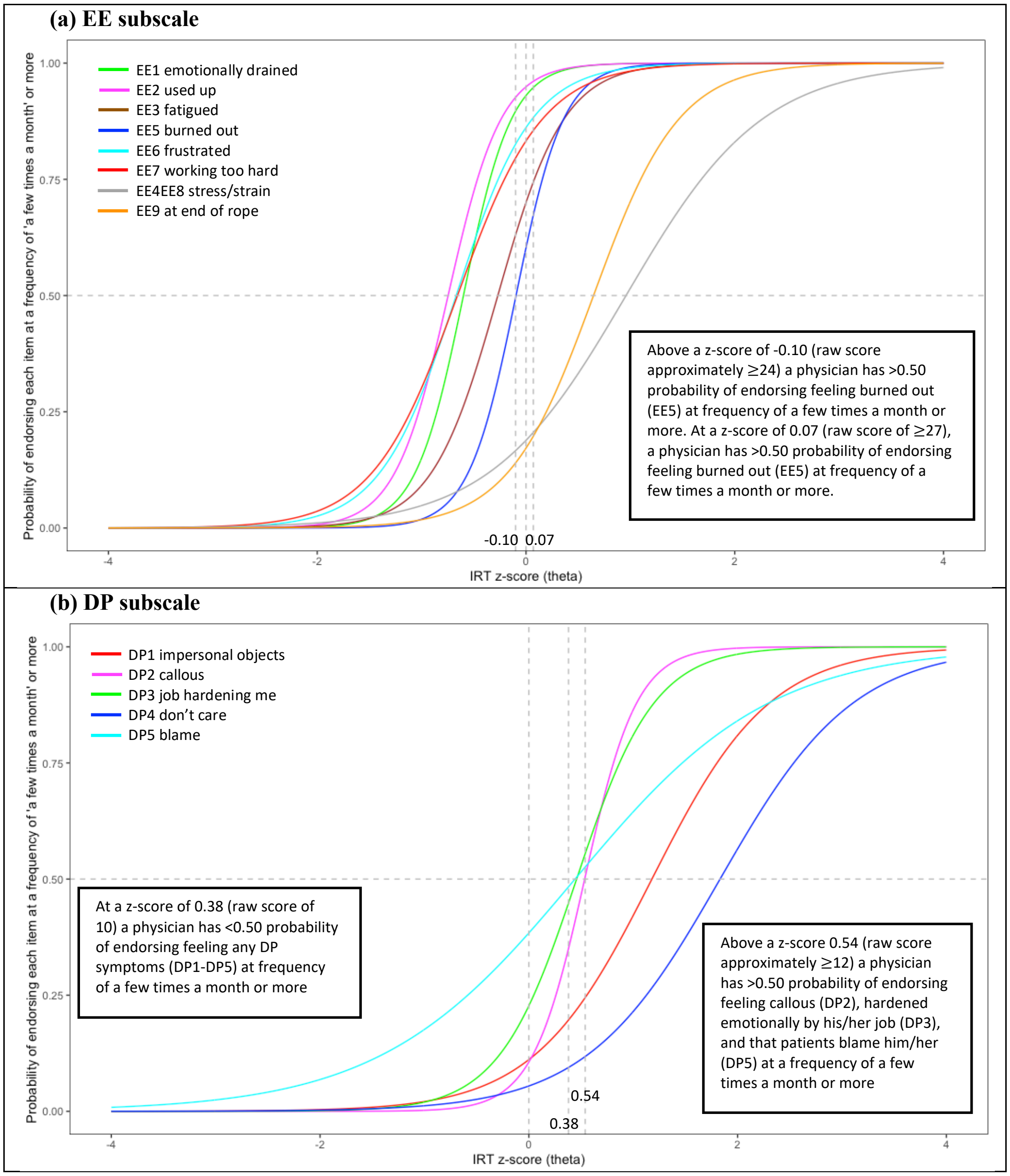

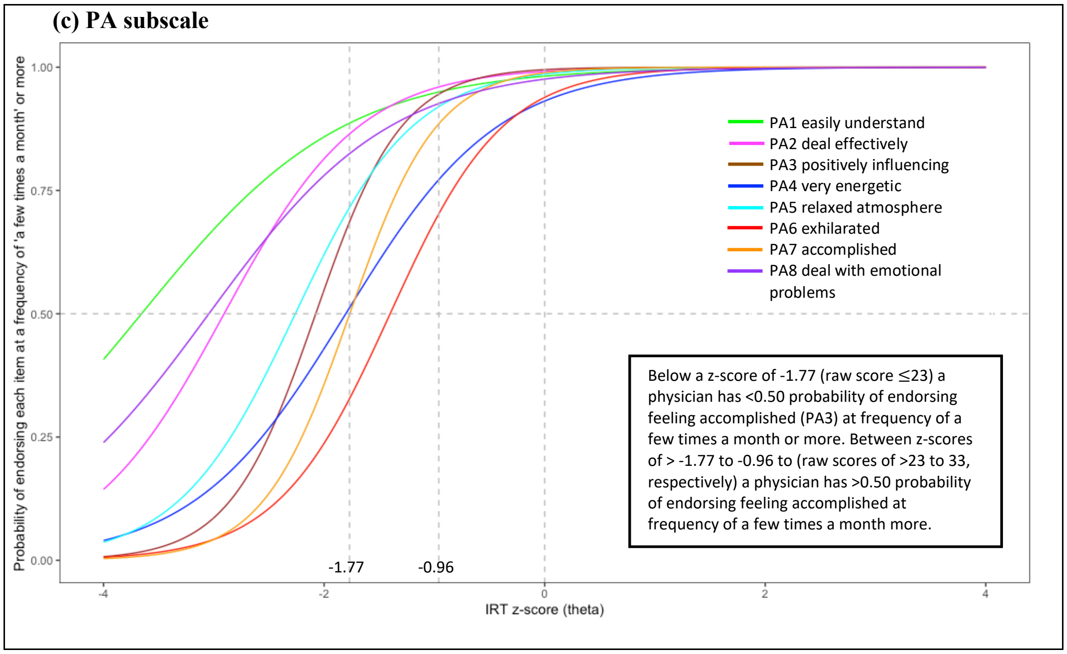

[a] Higher scores indicate higher EE, DP, and PA on each respective subscale. Higher scores on the EE and DP subscales indicate a higher burnout symptom burden. Lower scores on the PA subscale indicates a higher burnout symptom burden. EE and DP items located further to the right of the scale are items that represent a higher burnout symptom severity compared to items located further to the left of the scale. PA items located further to the left of the PA scale represent a higher burnout symptom severity compared to items located further to the right of the scale.

References

1. Reise, S. P., Morizot, J., & Hays, R. D. (2007). The role of the bifactor model in resolving dimensionality issues in health outcomes measures. *Quality of Life Research*, *16*(1), 19-31.
2. HealthMeasures. (2013). PROMIS® Instrument Development and Validation: Scientific Standards Version 2.0. http://www.healthmeasures.net/images/PROMIS/PROMISStandards_Vers2.0_Final.pdf
3. Rodriguez, A., Reise, S. P., & Haviland, M. G. (2016). Applying bifactor statistical indices in the evaluation of psychological measures. *Journal of personality assessment*, *98*(3), 223-237.
4. Rotenstein, L. S., Torre, M., Ramos, M. A., Rosales, R. C., Guille, C., Sen, S., & Mata, D. A. (2018). Prevalence of burnout among physicians: a systematic review. *Jama*, *320*(11), 1131-1150.
5. Strout, W. F. (1990). A new item response theory modeling approach with applications to unidimensionality assessment and ability estimation. *Psychometrika*, *55*(2), 293-325..
6. Rodriguez, A., Reise, S. P., & Haviland, M. G. (2016). Evaluating bifactor models: calculating and interpreting statistical indices. *Psychological methods*, *21*(2), 137.
7. Reise, S. P., & Revicki, D. A. (Eds.). (2014). *Handbook of item response theory modeling: Applications to typical performance assessment*. Routledge.
8. Reise, S. P., Moore, T. M., & Haviland, M. G. (2010). Bifactor models and rotations: Exploring the extent to which multidimensional data yield univocal scale scores. *Journal of personality assessment*, *92*(6), 544-559.
9. Muthén, B., Kaplan, D., Hollis, M. (1987). On structural equation modeling with data that are not missing completely at random. *Psychometrika*, *52*(3): 431-62.
10. Revelle, W. (2018). psych: Procedures for personality and psychological research. https://CRAN.R-project.org/package=psych.
11. Rosseel, Y. (2012). lavaan: An R package for structural equation modeling. *Journal of Statistical Software*, *48*(2): 1-36.
12. Hu, L. T., Bentler, P. M. (1999). Cutoff criteria for fit indexes in covariance structure analysis: Conventional criteria versus new alternatives. *Structural equation modeling: a multidisciplinary journal*, *6*(1): 1-55.
13. Steiger, J.H. (1990). Structural model evaluation and modification: An interval estimation approach. *Multivariate behavioral research*, *25*(2): 173-80.
14. Chen, F., Curran, P.J., Bollen, K.A., Kirby, J., Paxton, P. (2008). An empirical evaluation of the use of fixed cutoff points in RMSEA test statistic in structural equation models. *Sociological methods & research, 36*(4): 462-94.
15. Muthén, B., Du, S. H., Spisic, D., Muthén, B. O., & du Toit, S. H. C. (1997). Robust inference using weighted least squares and quadratic estimating equations in latent variable modeling with categorical and continuous outcomes. *Psychometrika*.
16. Satorra, A., Bentler, P.M. (2001). A scaled difference chi-square test statistic for moment structure analysis. *Psychometrika*; *66*(4): 507-14.
17. Xia, Y. (2016). Investigating the chi-square-based model-fit indexes for WLSMV and ULSMV estimators. https://diginole.lib.fsu.edu/islandora/object/fsu:366138/datastream/PDF/view
18. Reeve, B. B., Hays, R. D., Bjorner, J. B., Cook, K. F., Crane, P. K., Teresi, J. A., Thissen, D, Revicki, D. A., Weiss, D., J., Hambleton, R. K., Honghu, L., Gershon, R., Reise, S. P., Lai, J., Cella, D. (2007). Psychometric evaluation and calibration of health-related quality of life item banks: plans for the Patient-Reported Outcomes Measurement Information System (PROMIS). *Medical care*, S22-S31.
19. Chen, W. H., Thissen, D. (1997). Local dependence indexes for item pairs using item response theory. *Journal of Educational and Behavioral Statistics*, *22*(3): 265-89.
20. Chalmers, P. (2012). mirt: A multidimensional item response theory package for the R environment. *Journal of Statistical Software*, *48*(6): 1-29.
21. Rea, L.M., Parker, R.A. (2014). Designing and conducting survey research: A comprehensive guide: John Wiley & Sons.

22. Kazis, L. E., Marino, M., Ni, P., Bori, M. S., Amaya, F., Dore, E., Ryan, C.M. Schneider, J. C., Shie, V., Acton, A. & Jette, A. M. (2017). Development of the life impact burn recovery evaluation (LIBRE) profile: assessing burn survivors' social participation. *Quality of life research*, *26*(10), 2851-2866.
23. Samejima, F. (1969). Estimation of latent ability using a response pattern of graded scores. *Psychometrika monograph supplement*.
24. Maydeu-Olivares, A. (2014). Evaluating the fit of IRT models. Handbook of item response theory modeling: Routledge, 129-45.
25. Hays, R.D. (2011). Response 1 to Reeve's Chapter: Applying Item Response Theory for Questionnaire Evaluation. *Question evaluation methods: Contributing to the science of data quality*: 125-35.
26. Orlando, M., & Thissen, D. (2000). Likelihood-based item-fit indices for dichotomous item response theory models. *Applied Psychological Measurement*, *24*(1), 50-64.
27. Orlando, M., & Thissen, D. (2003). Further investigation of the performance of S-X2: An item fit index for use with dichotomous item response theory models. *Applied Psychological Measurement*, *27*(4), 289-298.
28. Kang, T., Chen T.T. (2011). Performance of the generalized S-X2 item fit index for the graded response model. *Asia Pacific Education Review*, *12*(1): 89-96.
29. Kang, T., & Chen, T. T. (2008). Performance of the generalized S-X2 item fit index for polytomous IRT models. *Journal of Educational Measurement*, *45*(4), 391-406.
30. Rose, M., Bjorner, J.B., Gandek, B., Bruce, B., Fries, J.F., Ware, J.E. (2014). The PROMIS Physical Function item bank was calibrated to a standardized metric and shown to improve measurement efficiency. *Journal of Clinical Epidemiology*, *67*(5): 516-26.
31. Reise, S.P. (1990). A comparison of item-and person-fit methods of assessing model-data fit in IRT. *Applied Psychological Measurement*, *14*(2): 127-37.
32. De Ayala, R.J. (2013). The theory and practice of item response theory: Guilford Publications; 2013.
33. Drasgow, F., Levine, M.V., Williams, E.A. (1985). Appropriateness measurement with polychotomous item response models and standardized indices. *British Journal of Mathematical and Statistical Psychology*, *38*(1): 67-86.
34. Felt, J. M., Castaneda, R., Tiemensma, J., & Depaoli, S. (2017). Using person fit statistics to detect outliers in survey research. *Frontiers in psychology*, *8*, 863.
35. Reise, S. P., & Waller, N. G. (2009). Item response theory and clinical measurement. *Annual review of clinical psychology*, *5*, 27-48.
